# The neighborhood social environment and physical activity: a systematic scoping review

**DOI:** 10.1186/s12966-019-0873-7

**Published:** 2019-12-09

**Authors:** Maura M. Kepper, Candice A. Myers, Kara D. Denstel, Ruth F. Hunter, Win Guan, Stephanie T. Broyles

**Affiliations:** 1 Prevention Research Center, Washington University in St. Louis, 1 Brookings Drive, St. Louis, MO 63130 USA; 2 Pennington Biomedical Research Center, 6400 Perkins Road, Baton Rouge, LA 70808 USA; 3 Queen’s University Belfast, University Road, Belfast, BT7 1NN UK; 4 Louisiana Department of Health, Bureau of Chronic Disease Prevention and Healthcare Access, 628 North 4th St., Baton Rouge, LA 70802 USA

**Keywords:** Physical activity, Social environment, Neighborhood, Scoping review

## Abstract

**Background:**

Investigating the association of the neighborhood social environment on physical activity is complex. A systematic scoping review was performed to (1) provide an inventory of studies assessing the influence of the neighborhood social environment on physical activity since 2006; (2) describe methodologies employed; and (3) formulate recommendations for the field.

**Methods:**

Two databases were searched using terms related to ‘physical activity,’ ‘neighborhood,’ and ‘social environment’ in January 2017. Eligibility criteria included: 1) physical activity as an outcome; 2) neighborhood social environment as a predictor; 3) healthy population (without diagnosed clinical condition or special population); 4) observational or experimental design. Of 1352 studies identified, 181 were included. Textual data relevant to the social environment measurement and analysis were extracted from each article into qualitative software (MAXQDA) and coded to identify social environmental constructs, measurement methods, level of measurement (individual vs. aggregated to neighborhood), and whether authors explicitly recognized the construct as the social environment. The following measures were generated for each construct: number of unique measurements; % of times measured at an aggregate level; % of times authors referred to the construct as the social environment. Social environmental constructs were then grouped into larger descriptive dimensions.

**Results/findings:**

Fifty-nine social environmental constructs were identified and grouped into 9 dimensions: Crime & Safety (*n* = 133 studies; included in 73% of studies); Economic & Social Disadvantage (*n* = 55, 33%); Social Cohesion & Capital (*n* = 47, 26%); Social Relationships (*n* = 22, 12%); Social Environment (*n* = 16, 9%); Disorder & Incivilities (*n* = 15, 8%); Sense of Place/Belonging (*n* = 8, 4%); Discrimination/Segregation (*n* = 3, 2%); Civic Participation & Engagement (*n* = 2, 1%). Across all articles, the social environment was measured using 176 different methods, was measured at an aggregate-level 38% of the time, and referred to as the social environment 23% of the time.

**Conclusions:**

Inconsistent terminology, definitions, and measurement of the social environment and the lack of explicit language identifying constructs as the social environment make it challenging to compare results across studies and draw conclusions. Improvements are needed to increase our understanding of social environmental correlates and/or determinants of physical activity and facilitate cross-disciplinary conversations necessary to effectively intervene to promote physical activity.

**Trial registration:**

PROSPERO CRD42017059580.

## Background

Physical activity is recognized as a healthy behavior that can prevent and treat numerous physical and psychological health conditions, including several chronic diseases such as obesity, diabetes, and cardiovascular disease [1]. Yet, most populations largely lead inactive lifestyles. Efforts to promote physical activity by health professionals (researchers, practitioners, and policy makers) are driven by an ecological approach that considers multiple levels (i.e. inter-personal, social, and physical environments, policies) that may influence an individual’s ability to achieve recommended levels of physical activity. There is broad agreement that the neighborhood environment must be considered for public health approaches to effectively improve physical activity [2]. The role of built environmental factors (e.g. access to greenspace and parks) on physical activity is largely recognized as evidenced by the Healthy People 2020 goals to include interventions targeting the built environment [3]. However, the role of the social environment is less clear despite its equal and potentially more prominent role in shaping physical activity [4].

The neighborhood social environment is defined as the sociodemographic composition of the neighborhood and its residents, as well as the relationships, groups, and social processes that exist among individuals living in the neighborhood [4–6]. In 2006, McNeill and colleagues [6] provided a taxonomy of dimensions of the social environment in an effort to bring clarity to the growing and evolving literature on the social environment and its influence on health behaviors. This taxonomy included five dimensions of the social environment—social support and social networks, socioeconomic positions and income inequality, racial discrimination, social cohesion and social capital and neighborhood factors—and described the mechanisms by which they influence behavior, particularly physical activity. Despite the continued growth of literature on the social environment and physical activity, McNeill’s 2006 taxonomy has not been systematically explored or updated. As argued by McNeill, a clear taxonomy is necessary to build and interpret a base of evidence for different social environmental characteristics and their relationships to physical activity.

To generate clarity on the neighborhood social environment, we sought to systematically identify and categorize aspects of the neighborhood social environment that were studied in association with physical activity since 2006. Furthermore, we built on McNeill’s review by focusing on the neighborhood social environment, rather than the social environment as a whole, and systematically assessed measurement of the social environment to expand on challenges and advancements in the field. A systematic scoping review [7] was performed to address three main objectives: (1) to provide an inventory of investigations of the neighborhood social environment on physical activity since McNeill’s summary of the literature in 2006; (2) to describe and critically discuss the methodological approaches employed in identified studies; and (3) to formulate recommendations for advancing the study of the neighborhood social environment on physical activity. Ultimately, the intent of this review is to grow the scientific understanding of how the neighborhood social environment is conceptualized and measured to accelerate this field of research and elucidate social environmental targets for interventions to promote physical activity and improve health.

## Methods

This systematic scoping review used the framework first presented by Arskey and O’Malley (2005) [7] and updated by several others [8, 9]. While scoping reviews follow a similar research process as systematic reviews, a scoping study addresses broad research topics where many different study designs are applied with the aim of comprehensively examining the extent, range, and nature of research activity in order to identify key concepts, results and gaps in the literature. Based on a review of research on scoping studies, our team deemed the scoping methodology appropriate for the examination of the neighborhood social environment and physical activity behaviors as this is an understudied, complex research area. Despite the broad, formative approach, scoping reviews can be conducted using systematic, explicit methods; therefore, we complied with established reporting guidelines for conventional systematic reviews to ensure transparency (PRISMA checklist, Additional file 1). The methods applied in this systematic scoping review were specified a priori and documented in a protocol developed by all members of the review team (Protocol PROSPERO CRD42017059580, https://www.crd.york.ac.uk/prospero/display_record.php?ID=CRD42017059580). Our application of the six-stage framework for performing a scoping study [7] is outlined below.

### Defining the research question (stage 1)

The main focus of this review was to gain a conceptual understanding of how researchers characterize and measure the neighborhood social environment when examining its effect on physical activity. This review builds on an existing review by McNeill and colleagues, which examined literature published prior to 2006 to provide a summary of the neighborhood social environmental dimensions and mechanisms specific to physical activity. This review updates McNeill’s definition of the neighborhood social environment and expands on methodological and conceptual challenges in order to advance the study of the neighborhood social environment on physical activity. We conclude with suggested recommendations for social epidemiologists and intervention researchers who seek to further understand social environmental determinants of physical activity.

### Identifying the relevant studies (stage 2)

One reviewer searched PubMed and EBSCOHost including: MEDLINE; CINAHL Complete; SOCINDEX; SPORTDISCUS using combinations of key search terms that reflected: environment, physical activity, and social constructs (see full search terms in Additional file 2). Social construct search terms included those identified as the social environment by McNeill and colleagues (2006) and by authors of the current review with expertise in the existing social environment literature. Articles published in English from January 2006 to January 2017 were included in order to expand on McNeill, et al.’s conclusions drawn from pre-2006 literature.

### Study selection (stage 3)

Articles were first screened for eligibility according to the title and abstract using standardized inclusion criteria (Table 1). For review articles that met inclusion criteria (*n* = 15), the reference lists were screened for additional articles. Article screening was independently performed by five reviewers. At the beginning of the title/abstract and full-text review, reviewers ensured consistency (i.e. consensus among all reviewers) by concurrently screening titles/abstracts and full-texts in sets of five for the first 20 articles (1% of abstracts and 10% of full-texts screened). Throughout the review process, any study flagged by a reviewer as uncertain for inclusion was discussed by all reviewers for eligibility.

**Table 1:** Inclusion criteria for the literature search

Term	Include
Study population	Healthy^a^ individuals of any age
Study design	Observational (cross-sectional and longitudinal designs), experimental (randomized controlled trials, pre-post designs, quasi experimental studies)
Predictor	Social environmental construct operating at or representing the neighborhood.^b^
Outcome	Physical activity^c^
Publication Date	Between January 1, 2006 to January 31, 2017
Language	English
Geography	Worldwide

^a^ Healthy was defined as an individual without a diagnosed clinical condition (e.g., diabetes, cardiovascular disease, cancer, asthma) and that is not classified as a special population. Individuals/populations with overweight/obesity were included

^b^ Workplace or institutional social environmental measures were not included

^c^All measures of physical activity were included, regardless of validity and/or reliability of the methods

### Interpreting and synthesizing or charting the data (stage 4)

Data were extracted using a standardized electronic form. The following data were extracted from each paper: study design, study location, study setting (urban, rural, or both), sample size, percent male, age range, and whether physical activity was subjectively- or objectively-measured. Sample age was categorized as youth (≤18 year of age), adult (> 18 year of age), or both. Physical activity was considered to be objectively-measured if a device (i.e. accelerometer, pedometer, heart rate monitor, etc.) was used to measure physical activity. Subjectively-measured physical activity included self- or parent-reports using a survey instrument.

In addition to extracting these summary data, each reviewer extracted all text relevant to the social environment measurement and analysis. Extracted text for each article was uploaded into qualitative analysis software (MAXQDA Analytics Pro, VERBI Software, 2017) and coded to capture all social environmental constructs. In addition to the overall goal, we aimed to capture: 1) social environmental constructs even when authors did not acknowledge them as the social environment and 2) how authors were referring to/defining the social environment. Therefore, deductive and inductive methods were used to establish a codebook. Social environmental constructs identified by prior published reviews [4, 6] were included as deductive codes, regardless of whether or not the manuscript authors acknowledged the construct as a measure of the social environment. Codes were added inductively if authors identified a new construct as the social environment. Additionally, when authors measured a previously described deductive social environmental construct using different or “new” terminology, an inductive code was created that corresponded to the manuscript authors’ terminology.

Codes were also established to capture details on measurement, level of measurement (individual vs. aggregate), and whether or not the author explicitly recognized the construct as the social environment. Aggregate was defined as a measure performed at an area-level (e.g., census characteristics for a census tract or block group) or the use of methodological approaches to aggregate the responses of raters or survey respondents to characterize an area [5]. Measures that assessed an individual’s environment or area (e.g., disorder on the street in which an individual lived or an individual’s perceived neighborhood characteristics) were considered individual variables. Approximately 20 % of the articles (*n* = 36) were coded by two raters to assess inter-reviewer reliability. Inter-reviewer reliability was established as the percent agreement between two raters for the presence and frequency of the same codes within the document. This measure was chosen to confirm that reviewers were identifying the same social environmental variables within articles and correctly determining the constructs level of measurement (individual vs. aggregate).

### Collating summarizing and reporting the results (stage 5)

The full list of 176 unique social environmental codes/constructs was grouped into larger social environmental dimensions. Four reviewers independently grouped similar codes and assigned descriptive names for each group. Discrepancies were discussed to generate a consensus among all reviewers. Once dimensions were created, reviewers systematically examined how each social environmental construct was measured to determine the number of unique measurement methods used for each construct. A single study may have included multiple social environmental constructs, and single construct may have been measured multiple ways in a single study (e.g. disadvantage measured as annual household income at the individual-level and aggregated to neighborhood income in the same study). Across studies including a specific social environmental construct, the following summary measures were calculated: 1) the percent of times the construct was measured at an aggregate level and 2) the percent of times authors referred to the construct as the social environment.

## Results

### Study selection

**Fig. 1:**
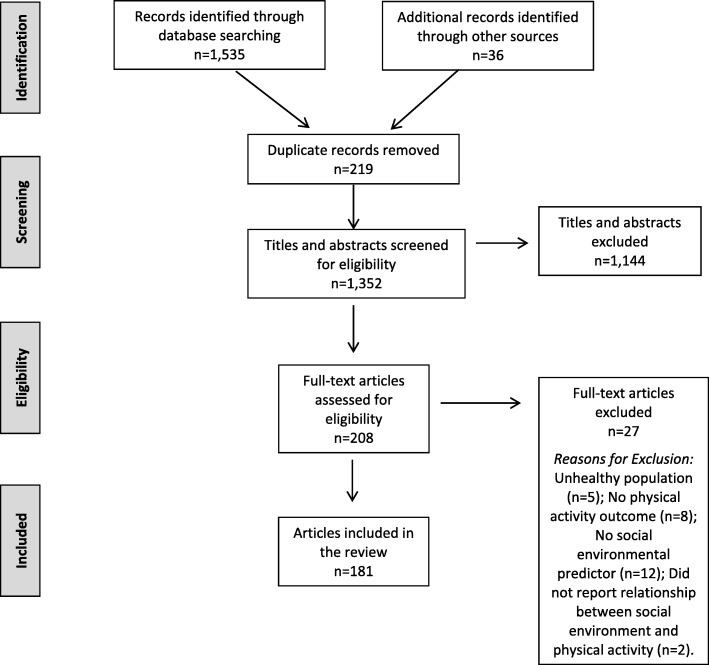
PRISMA flowchart for study selection (stage 3)

Our search yielded a total of 1352 articles after removal of duplicates (*n* = 219) and inclusion of articles identified from the reference lists of 15 review articles (*n* = 36). Of these 1352 articles, 1144 were excluded from our review based on title and abstract review. Full-text review (*n* = 208) resulted in a total of 181 articles included in this review (Fig. 1).

### Study characteristics

A total of 181 articles were reviewed and coded. Inter-reviewer reliability for coding was greater than 95% for each code with an average of 99.3%. An overview of the characteristics of each included study is provided in Additional file 3: Table S1. A summary of study characteristics is reported in Additional file 4: Table S2. Most studies were cross-sectional (91.7%), conducted in adults (58.5%), and performed in urban settings (58.0%). Sample sizes ranged from 27 to 964,318 with a median of 1055 participants. Studies were performed worldwide, with the majority in North America (51.9%) and Europe (25.4%). Objectively-measured physical activity was included in 17% of articles. The social environment was characterized using 59 different constructs. Of these constructs, 37 (63%) were identified inductively.

### Measures of physical activity

There was variability across articles in the methods of assessing physical activity, the way in which cited questionnaires were used, modified, or adapted, and the dimensions of physical activity measured (i.e., intensity, total physical activity, walking behavior, active transportation, sports participation, etc.). The majority of articles relied on self- or parent-report measures (i.e., questionnaires) to assess physical activity (*n* = 150, 82.8%). Commonly-used questionnaires were the 7- or 3-Day Physical Activity Recall (PAR); International Physical Activity Questionnaire (IPAQ); Godin and Shephard Instrument; Neighborhood Physical Activity Questionnaire (NPAQ); and items from the Behavioral Risk Factor Surveillance System (BRFSS). Of those articles that measured physical activity objectively (*n* = 31), 26 (83.9%) used accelerometers to assess mean daily activity counts per minute; minutes of moderate to vigorous physical activity (MVPA), light activity or sedentary behavior; and/or adherence to guidelines (e.g., ≥150 min of MVPA per week for adults). Three other types of devices were used to measure physical activity or walking objectively: 1) Global Positioning System (GPS) devices (*n* = 1) [10], which applied an algorithm to identify walking trips; 2) heart rate monitors (*n* = 1) [11] in conjunction with walking logs to examine walking frequency, duration and intensity; and 3) pedometers (*n* = 3) [12–14].

### Measures of the social environment by dimensions

**Fig. 2:**
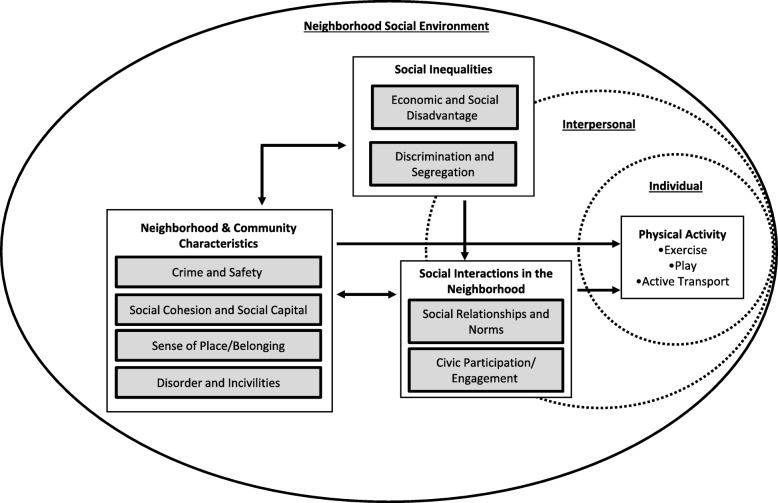
Conceptual framework for how the neighborhood social environment is related to [individual-level] physical activity. Adapted from McNeill et al. (2006) *Soc Sci Med* and Suglia et al. (2016) *J Urban Health*. Grey boxes indicate neighborhood social environment dimensions. Neighborhood measures can either be objectively/directly measured or perceived by individuals

Results are presented within nine dimensions which were determined by authors of this review and conceptually grouped into a framework with three domains (Social Inequalities, Neighborhood and Community Characteristics, and Social Interactions in the Neighborhood). This framework illustrates how the neighborhood social environment is conceptualized in relation to physical activity (Fig. 2). Each dimension is comprised of related social environmental constructs that were identified deductively or referred to by study authors (Additional file 5: Table S3).

### Social inequalities

#### Economic and social disadvantage

A total of 55 studies (30%) included a measure of economic and social disadvantage as a measure of the neighborhood social environment. The constructs were referred to by study authors as affluence (*n* = 1), education (*n* = 4), income (*n* = 5), income distribution (*n* = 1), income inequality (*n* = 2), poverty (*n* = 7), socioeconomic status (SES; *n* = 21), deprivation (*n* = 17), disadvantage (*n* = 4), social development (*n* = 1), and structural characteristics (*n* = 1). Only 12% of the time did the authors explicitly refer to the construct as a measure of the social environment. Across the 55 studies that included social environmental constructs in this dimension, 42 different measurements were used, and most measures corresponded to an aggregate-level (98% of the time). Constructs in this dimension were commonly-measured using census data to characterize economic or social disadvantage for the census tracts in which study participants lived.

Neighborhood SES was the most frequently measured construct in this dimension, and was measured in various ways. Several studies [15–17] used median household income of a census tract to measure neighborhood SES. Three studies in Australia used the Index of Relative Socio-Economic Advantage/Disadvantage (IRSD) to measure neighborhood SES [18–20]. Three other articles used the IRSD but identified this measure as neighborhood disadvantage [21–23]. Other studies measured neighborhood SES using scales or factors created from aggregate data on unemployment, income, education, or homeownership. Indices or factors that authors defined as deprivation (the second most frequently measured construct in this dimension) included comparable or the same variables as those used in neighborhood SES measures. The Townsend Index [24] was used by three articles in this dimension, two of which referred to the construct as deprivation [25, 26] and one identified it as neighborhood SES [27].

#### Discrimination and segregation

A total of three studies (2%) included a measure of Discrimination and Segregation [28–30]. This dimension included two constructs that authors referred to as racism (*n* = 1) and racial segregation (*n* = 2). Two (67%) of the authors referred to these constructs as the social environment. The Discrimination and Segregation dimension was measured at the aggregate level 75% of the time. Racial segregation was measured as the percentage of African Americans in a census tract. The method for determining which tracts were considered racially segregated was different between the two articles. Armstrong-Brown defined segregated as a census tract with 50% or more African American residents [29]. Whereas, Borrell et al., generated sample-specific tertiles of % African American in the census tract [30]. Community racism was measured at the individual-level by a single survey item that asked participants “How much of a problem is racism in the particular town, city, or rural area where they currently live?” [28].

### Neighborhood and community characteristics

#### Crime and safety

The Crime and Safety dimension included seven constructs that were included in 133 studies (73%) and measured using 31 different methods. The constructs were referred to by study authors as neighborhood danger (*n* = 1), crime (*n* = 48), safety (*n* = 95), stranger danger (*n* = 2), violence (*n* = 4), risk (*n* = 1), and environmental barriers (*n* = 2). Only 11% of the time were these constructs explicitly referred to as the social environment. The majority of articles (*n* = 128, 96%) in this dimension included safety (*n* = 95, 71%) and/or crime (*n* = 48, 36%). Crime was measured at the aggregate level 52% of the time compared to only 6% of the time for safety measures. Crime was predominantly measured in two ways: 1) using police reported crime data to ascertain aggregate/neighborhood levels of crime; and/or 2) self-reported perceived crime obtained via questionnaires. Crime measured using police-reported crime data were considered the same method of measurement; however, there was variation in the type of crimes included (e.g. violent crimes, thefts, and assault/rape) across the articles. Safety was predominantly measured as an individual’s perception (i.e., perceived safety from crime or traffic) using questionnaires. The Neighborhood Environment Walkability Survey (NEWS) was the most commonly-used questionnaire to measure perceived safety from crime and/or traffic (*n* = 27 articles, 28%). To measure perceived safety, the NEWS asks individuals to indicate the degree to which they agree or disagree on a 5-point Likert scale to six statements (e.g., “my neighborhood streets are well lit at night;” “there is a high crime rate in my neighborhood;” “the crime rate in my neighborhood makes it unsafe to walk at night”). Other questionnaires used to measure safety included the Environmental Module of International Physical Activity Prevalence Study, the Neighborhood Environment Scale, the Physical Activity Neighborhood Environment Survey (PANES), and the IPAQ.

#### Social cohesion and social capital

A total of 47 studies (26%) included at least one of the nine constructs that comprise the Social Cohesion and Social Capital dimension. These constructs were measured 40 different ways and were referred to by authors as social capital (*n* = 16), social cohesion (*n* = 25), collective insecurity (*n* = 1), trust (*n* = 4) collective efficacy (*n* = 3), hostility or distrust (*n* = 1), psychosocial aspects (*n* = 1), norms of reciprocity (*n* = 1), and social control (*n* = 1). These constructs were measured at an aggregate level 28% of the time and were referred to by the author as the social environment 43% of the time. Social cohesion was the most frequently measured variable in this dimension (*n* = 26, 55%) and was measured in 19 different ways. Eight of the articles [31–38] used the 5-items that measure social cohesion from the 10-item scale adapted from the Project on Human Development in Chicago Neighborhoods (PHDCN) study [39]. Several articles (*n* = 5, 19%) modified this social cohesion scale by conducting a factor analysis [40, 41], generating categorical variables (i.e., quartiles) [15, 42], or only using certain questions from the scale [43]. One article [44] only included social control, measured using the 5-items from the 10-item PHDCN study scale, rather than including both social control and cohesion [39]. All three collective efficacy (defined as social cohesion and control) measures were measured using all 10-items from this scale [39] and were at the individual-level [45–47].

#### Sense of place/belonging

A total of 8 articles (4%) included a measure of Sense of Place/Belonging, all of which used a different measurement. This dimension included 6 constructs that were referred to by study authors as place attachment (*n* = 2), sense of community (*n* = 1), territoriality (*n* = 1), feeling at home in one’s neighborhood (*n* = 1), social fragmentation (*n* = 1) and belonging (*n* = 1). Of the 8 times these constructs were measured, 3 (38%) were at measured at the aggregate level and 4 (50%) were clearly stated by the author as the social environment. Questionnaires were commonly used across this dimension. Both place attachment measurements were summary variables from multiple questionnaire items (e.g. how important it was for the participant to live in the particular neighborhood, if the neighborhood was a good place for children to grow up and thrive, expectations for living in the neighborhood for a long time, feeling at home in the neighborhood, involvement in the neighborhood) used to assess an individual’s perception of their neighborhood [44, 48]. Territoriality and Social Fragmentation were measured at the aggregate-level using neighborhood observation methods [49] and census data [50], respectively.

#### Disorder and incivilities

A total of 15 studies (8%) included one of the seven constructs in the Disorder and Incivilities dimension. The study authors referred to these constructs as incivilities (*n* = 2), disorder (*n* = 7), dog dirt or waste/litter (*n* = 2), unoccupied housing (*n* = 2), problems (*n* = 3), territoriality (*n* = 1) and social disorganization (*n* = 1); half of the time (55%) authors explicitly referred to these constructs as the social environment. These constructs were measured at an aggregate level 55% of the time. This dimension was commonly-measured using Systematic Social Observation (i.e. the Neighborhood Inventory of Environmental Typology Method, The Pedestrian Environment Data Scan; Neighborhood Observational Checklist), a method where raters systematically observe specific factors (i.e. litter, building condition, and presence of a sidewalk) in the neighborhood [46, 49, 51–53]. Other studies asked respondents whether individual’s perceived items of disorder/incivilities to be a problem in their neighborhood or whether they observe them in their environment [32, 37, 50, 54–56]. Disorder was operationalized as both physical disorder (e.g. presence of graffiti, garbage/litter on the street; vacant lots in poor condition; abandoned cars on the street; residential grounds in poor condition) and/or social disorder (e.g. presence of police or security guards, people loitering, congregating, or hanging out; people selling illegal drugs, drinking alcohol or smoking openly). Two of the studies [46, 51] used a modified version of the block physical disorder and physical decay measures created by Sampson and Raudenbush (2004) [57]. Another study [50] used 7 questions adapted from Sampson and Raudenbush (2004), yet these responses were combined to create a social disorder score for each individual. Three studies referred to disorder/incivilities as “neighborhood problems” [15, 31, 58].

### Social interactions in the neighborhood

#### Social relationships and norms

Twenty-two articles (12%) included a measure of Social Relationships and Norms as a measure of the neighborhood social environment. The constructs (*n* = 14) were referred to by study authors as social norms (*n* = 3), social networks (*n* = 1), social contacts (*n* = 1), neighborhood experiences (*n* = 1), social connectedness (*n* = 2), social support (*n* = 5), social ties (*n* = 1), social participation (*n* = 3), interpersonal relationships (*n* = 1), social interaction (*n* = 2), socializing (*n* = 1), social contacts (*n* = 1), social relations (*n* = 1), other children in neighborhood (*n* = 1). Across the 22 articles, 25 different measurements were used and were only measured at an aggregate-level 15% of the time. Authors explicitly recognized constructs in this dimension as the social environment half of the time (50%). Social support, the most common construct (*n* = 5), was measured using different questionnaires such as the Duke Social Support Scale [59]; six 4-item subscales that measured Reliable Alliance, Attachment, Guidance, Nurturance, Social Integration, and Reassurance of Worth [60]; a six item emotional support index [35]; the Family and Friend Support for Exercise Habits scale [53] and a tangible social support scale [44]. Social connectedness was measured two ways: by asking participants the number of neighbors that they knew by name [42] or the degree to which they agree with the statement ‘people in this neighborhood can be trusted.’ [61] Measures within this dimension that were at the aggregate level included: ‘Other Children in the Neighborhood’ measured as the population density of children [17] and ‘Social Relations’ measured using individual responses to Sampson’s neighborhood social cohesion and social control scale aggregated to the tract-level [62] and as individual’s social participation in different activities (e.g. recreational activities involving other people, activities of other political organizations; dining out or shopping with others) aggregated with other individual’s in the same neighborhood or municipality [63, 64].

#### Civic participation/engagement

A measure of Civic Participation/Engagement was included in 2 studies (1%) that used different measurements [48, 65]. This dimension included two constructs that were referred to by study authors as civic participation (*n* = 1) and civic engagement (*n* = 1). Civic participation [65] was measured at an aggregate-level (individual survey responses aggregated to census-tract level), whereas survey responses for community engagement [48] were treated as an individual-level construct. Community engagement, but not civic participation, was acknowledged by the author as a measure of the neighborhood social environment.

#### Social environment

This dimension included 16 studies (8%) in which the authors referred to the independent or predictor variable generically as the social environment. Aggregate measures were used 13% of the time. Methods used in this dimension were similar to or the same as those used in other dimensions. However, this dimension remained on its own because authors referred to the independent or predictor variable as the social environment. Seven articles assessed the social environment using one question (seeing people being active) from the IPAQ environmental module [66–72].

## Discussion

**Fig. 3:**
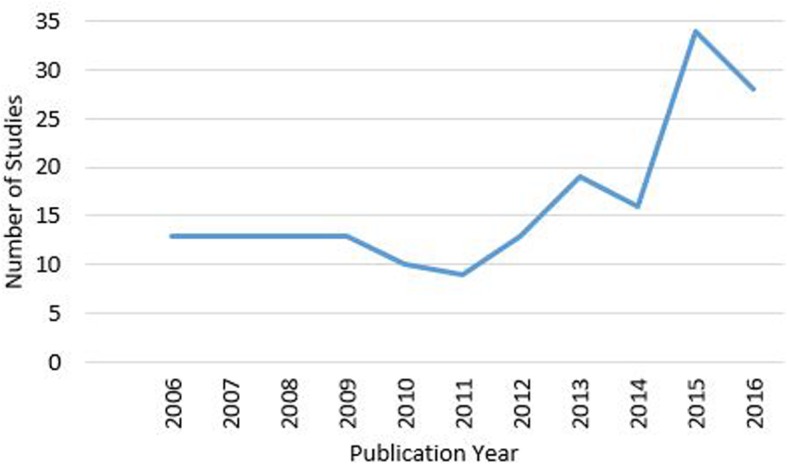
Number of studies examining the neighborhood social environment and physical activity over time

This systematic scoping review found 181 articles published since 2006 that assess the relationship between the neighborhood social environment and physical activity. Figure 3 illustrates that this is a growing area of research. An earlier review by McNeill et al. (2006) provided a taxonomy of social environmental dimensions to bring clarity to this literature [6]. The current scoping review extends our prior knowledge by systematically examining how the neighborhood social environment is defined, operationalized and measured in relation to physical activity. This review finds that, despite the taxonomy provided by McNeill et al., social environmental constructs are assessed and defined inconsistently, which creates challenges in synthesizing this literature. Moreover, authors frequently fail to explicitly refer to neighborhood social environmental constructs as such, further limiting our ability to identify important social environmental influences that may be modified to promote physical activity. This review updates a conceptual framework for how the neighborhood social environment may influence physical activity and provides specific recommendations in order to guide future research and improve the evidence.

This scoping review finds that, with respect to physical activity, nine dimensions of the neighborhood social environment have received attention in the literature. The most commonly-examined dimensions were Crime and Safety (*n* = 133 studies, 73%) and Economic and Social Disadvantage (*n* = 55 studies, 30%). Our findings are in line with a previous review which found that neighborhood poverty is the most frequently examined neighborhood social characteristic [4]. Furthermore, the environmental subgroup of the Accumulating Data to Optimally Predict obesity Treatment (ADOPT) Core Measures Project recognized neighborhood socioeconomic environment and crime-related safety as social environmental factors for which there is substantial evidence for consistent associations with weight-related behaviors and high measurement feasibility [73]. ADOPT also identified Social Relationships and Norms (*n* = 22, 12%; termed in ADOPT as individual social norms and social support) as an area with sufficient evidence and measurement potential [73]. This review identified additional social environmental dimensions not recognized by ADOPT or McNeill (2006) that may have important implications for physical activity. The new dimensions include: Sense of Place/Belonging; Disorder and Incivilities; and Civic Participation/Engagement. Since 2006, the definition and conceptualization of the social environment has grown. Our conceptual framework (Fig. 2) provides evidence-based, conceptualization and terminology that researchers may use to build consistency in the field and grow our understanding of social environmental influences on physical activity.

Dimensions included multiple social environmental constructs due to the use of inconsistent terminology and measurement across studies. The majority of neighborhood social environmental constructs (63%) were unique from predetermined constructs or other studies’ terminology. For example, within the Economic and Social Disadvantage dimension, authors used 11 different constructs, of which four were added inductively. Constructs within this dimension were measured using 42 methods. Across all dimensions, 176 different methods were used to measure the neighborhood social environment. As recognized previously by Diez Roux et al. [5], the explosion of measurement techniques and measures signifies advancements in studying how the neighborhood social environment influences physical activity, but it also creates challenges. Inconsistent terminology and diverse measures limit the ability to synthesize the evidence, understand the impact of the social environment on physical activity, identify targets for interventions and elicit change from policy makers and other stakeholders. This review provides a platform from which the field can develop cohesive language and identify rigorous measures. As advocated for in built environment research, consolidating the long list of constructs is a key step toward widespread use of rigorous measures and efficient analysis to synthesize and understand the state of the evidence. The use of the developed framework to promote a common language when appropriate is the first step. Two additional steps are necessary for further consolidation: 1) critically assess measures used for the nine neighborhood social environmental dimensions identified in this review, as was done for the built environment [74] and 2) determine the magnitude of association between social environmental constructs and physical activity to further consolidate key measures in the field.

The neighborhood is an area that bounds social processes, yet measures of the social environment were rarely at an aggregate- or area-level. Across all studies, constructs were measured at an aggregate-level 38% of the time. Certain dimensions (i.e. Economic and Social disadvantage, Discrimination and Segregation, and Disorder and Incivilities) were more likely to use aggregate-level measures, as these dimensions are easier to assess using publically-available data (e.g. United States Census Bureau) or neighborhood observation methods (e.g. Systematic Social Observation) that make aggregate-level measures more feasible. This finding is in line with a systematic review on neighborhood effects research, which found that the majority of studies (43.2%) used census-based variables [75]. Dimensions that relied on study-specific survey measures (i.e. Social Relationships and Norms; Sense of Place/Belonging) were less likely to aggregate responses or identify alternative aggregate-level measurements. As argued by Sampson & Raudenbush (1999) at the start of neighborhood effects research, measurement of an ecological or social unit cannot rely solely on the tools of psychometrics. Progress in social environmental effects research has been made; yet, methodological challenges (i.e. bias, sample size, the area-definition, etc.) remain and require a greater understanding to improve aggregate measures and increase their use when appropriate [76, 77]. Defining neighborhood boundaries is the most commonly-cited challenge as it varies based on the hypothesized causal processes [5, 75, 78]. For example, smaller geographic areas may be more pertinent to social relationships among neighbors, whereas larger areas may be appropriate for crime and safety. Qualitative studies that examine how individuals interact with varying spatial contexts and technology (i.e. Geographic Information Systems [GIS], GPS with Accelerometry) that allow objective assessment of spatial patterns of behavior are striving to understand appropriate definitions of exposure. On a distinct but related point, such technologies can derive outcome measures that are “conceptually-matched” to a spatial exposure (i.e. within-neighborhood physical activity), which encourages more rigorous inferences [79, 80]. Yet, these technologies are rarely used. Studies in this review largely used subjective measures of physical activity, which may be biased as individuals often overestimate physical activity [81]. Furthermore, this review found that authors rarely acknowledge constructs as the social environment which may signify that consideration of theoretical models underlying the research questions and purposeful selection of measurement is lacking. Ultimately, careful consideration of the causal processes with attention to how specific neighborhood attributes may be related to health and behavior remains a priority to advance measurement and improve our understanding [5].

### Methodological considerations and recommendations for future research

Table 2 details a number of methodological issues raised in this review and provides recommendations to improve the evidence for neighborhood social environmental effects on physical activity. Employing different terminology to define constructs and the lack of explicit language identifying the construct as the social environment makes it challenging to compare results across studies and draw general conclusions [82]. The 59 neighborhood social environmental constructs in this review are not unique concepts, rather they reflect the multitude of ways in which researchers are referring to overlapping social environmental constructs. We recommend researchers and practitioners standardize the terminology used when it is appropriate to provide consistency in how neighborhood social environmental constructs are defined (Recommendation 1). The dimensions generated from this review represent unique social environmental concepts that provide a foundation from which authors may choose to use common terms that are appropriate for the social environmental concept of interest. Our hope is that the multitude of ways in which a single social environmental concept was termed reduces with the use of the established framework. A consistent language will eliminate unnecessary complexity that burdens our understanding of the importance of the social environment on physical activity, as well as other behaviors and health outcomes. When a study includes one of the neighborhood social environmental dimensions identified in this review, authors should explicitly state that they are examining the social environment (Recommendation 2). Identifying constructs as the social environment will provide context for theoretical underpinnings, and increase a study’s ability to be discovered and synthesized by others in order to draw more powerful conclusions on social environmental correlates and/or determinants of physical activity. Based on the large number of measures used across articles in this review, there is a clear need for guidance on rigorous measures to improve measurement precision and standardize methods across studies. It is important to acknowledge that there may be instances when standardization of terminology and methods is not appropriate (e.g., does not align with the research question or causal processes), yet we are advocating for the field to purposively select the term and measurement used for the social environmental concept with consideration of the framework established from this review. When using a common-method or tool, researchers should use caution when altering aspects of the approach, including data collection (e.g., selecting certain questions in a scale) and analysis (e.g., using factor analysis or generating categorical variables) that may make results more difficult to compare with other studies using the same method/tool (Recommendation 3). Measurements in this field should consider the appropriate spatial context. Exposure areas (i.e., neighborhood definition) should be based on the environmental construct, health behavior, and outcome of interest as well as the hypothesized causal processes involved (Recommendation 4). We advocate for the continued use of methods well-suited for the study of environmental health effects (i.e., multilevel statistical analysis, spatial analysis techniques, and spatial software such as GIS) while striving for consistency in measurement and terminology. Furthermore, our results illustrate a dominance of cross-sectional study designs, which have been acknowledged as a challenge to understanding neighborhood effects on health behaviors and outcomes. Challenges with cross-sectional designs include confounding by unobserved co-variates, structural confounding and the related threat of generating off-support estimates, and reverse causation [5, 75, 77, 83]. Leaders have long called for diverse study designs, such as longitudinal designs following people as they transition between neighborhoods and as neighborhoods evolve around them and intervention studies designed to inform place-based efforts to improve health [78]. A 2016 review of neighborhood effects research reported little movement toward diverse study designs [75]. However, a recent review that aimed to identify which built environmental interventions increase physical activity found 28 studies that applied these designs where causality can be implied [84]. Our results indicate that few studies examining the neighborhood social environment for physical activity apply these designs, which demonstrates a clear need for longitudinal and quasi, natural or fully experimental research (Recommendation 5).

**Table 2:** Summary of recommendations for future research

	Methodological Issue	Recommendation	Anticipated Improvements
1	Inconsistent terminology	Standardize terminology of the neighborhood social environmental using dimensions identified in this review (Fig. 2) when appropriate.	Compare results across studies. Synthesize the evidence. Increased understanding.
2	Identification of the social environment	Clearly indicate that the construct is a neighborhood social environmental construct.	Recognize research studies exploring the phenomenon. Compare results across studies. Synthesize the evidence. Increased understanding.
3	Abundant measures	Use measurement tools and methods that are specific, rigorous and validated for the neighborhood social environmental construct of interest. Standardize measurement methods in the field. When using common methods/measurement tools be cautious about altering the approach through data collection (e.g. selecting only certain questions of a scale) or analysis (e.g. using factor analysis or generating categorical variables) that may make your results more difficult to compare.	Compare results across studies. Synthesize the evidence. Increased understanding.
4	Level of measurement	When appropriate, employ measurement strategies that facilitate neighborhood-level measurement. Use neighborhood definitions that are specific to your hypothesis (i.e., environment exposure, outcome and causal processes)	Improved rigor of measurement. Increased understanding.
5	Study design	Use of diverse study designs (e.g. longitudinal and quasi, natural or fully experimental research).	Increased ability to determine causality. Increased understanding.

### Limitations

A major strength of this systematic scoping review is its broad scope. Our application of innovative text mining methods and use of qualitative software allowed us to apply a systematic, yet comprehensive, assessment of how current literature conceptualizes and measures the neighborhood social environment in relation to physical activity. The significance and effect size of social environmental factors on physical activity was not explored. Therefore, no conclusions can be drawn as to the magnitude of associations between physical activity and various social environmental constructs and measurements. We did not examine the strengths or weaknesses of the different measures used and our review was not designed to determine if certain constructs better represent the dimension as a whole. Similar to the review performed for the built environment and physical activity [74], a critical review of measurement tools and methodologies is needed for the social environment. These limitations specify vital next steps that are necessary to build upon this review to establish consistency, rigor and, ultimately, better understanding in the field.

The aim of this review was to examine neighborhood social environmental constructs; yet, studies that assessed the social environment at the community-level, rather than being specific to the neighborhood, may have been included. This limitation is related to the previously discussed methodological challenge regarding the heterogeneity of neighborhood definitions. Furthermore, the problem may stem from ambiguity in terminology (e.g. community vs. neighborhood) used by study authors or within surveys that may elicit responses that are not specific to the neighborhood. However, the conclusions of this review (i.e. dimensions and conceptual framework) are not likely to be affected by this limitation. Furthermore, we did not determine whether the same method was used to measure social environmental constructs that were conceptually the same but referred differently by authors. Therefore, when we summed the number of measures used for all constructs within a dimension we may have over-represented the number of unique measures within and across dimensions. For example, if ‘% of households below the poverty-level in a census tract’ was used to measure both SES and poverty, two separate constructs within the Economic and Social Disadvantage dimension, it would have been counted as two measures within the dimension, ultimately overestimating the total number of unique measures within the dimension. This review included safety as a social environmental construct. Our original intent was to exclude safety from traffic, as traffic was considered a physical environmental factor. However, differentiating between safety from crime and safety from traffic was challenging, as they were often times measured or analyzed together. This differentiation should be considered in the field, as identifying how to intervene to change perceptions of safety may differ based on what factors are contributing to an individual’s perception. In other words, do we need to intervene to reduce crime, traffic, or both to improve people’s perceptions of safety in their neighborhood? For the purposes of this review, if perceived safety from crime and traffic were analyzed together it was included as safety; if only perceived safety from traffic was assessed the article was not included. Lastly, we cannot be certain that all articles examining the relationships between the social environment and physical activity were identified despite the comprehensive search strategy employed. As we concluded from this review, authors are using many terms and often fail to reference the social environment, therefore, our search may have overlooked an article.

## Conclusion

This review highlights the need to further understand which social environmental factors are key influencers of physical activity. Key steps to this understand are the use of appropriate terminology, explicit language, and rigorous measures for neighborhood social environmental constructs. The recommendations presented in this review are essential next steps to improve our understanding of the impact of the neighborhood social environment on physical activity and facilitate cross-disciplinary conversations to effectively intervene on neighborhood social environments to increase physical activity. This is critical as physical inactivity in a major public health concern, with substantial implications for our health, society and economy [85].

## Supplementary information

**Additional file 1.** PRISMA 2009 Checklist.

**Additional file 2.** Full Search Terms.

**Additional file 3: Table S1.** Overview of study characteristics.

**Additional file 4: Table S2.** Characteristics of articles reviewed (*n*=181).

**Additional file 5: Table S3.** Summary of social environmental constructs and measurements used in 181 published studies on physical activity.

## Data Availability

Not applicable.

## Abbreviations

ADOPT: Accumulating Data to Optimally Predict obesity Treatment
BRFSS: Behavioral Risk Factor Surveillance System
GIS: Geographic Information Systems
GPS: Global Positioning System
IPAQ: International Physical Activity Questionnaire
IRSD: Index of Relative Socio-Economic Advantage/Disadvantage
MVPA: Moderate to vigorous physical activity
NEWS: Neighborhood Environment Walkability Survey
NPAQ: Neighborhood Physical Activity Questionnaire
PANES: Physical Activity Neighborhood Environment Survey
PAR: Physical Activity Recall
PHDCN: Project on Human Development in Chicago Neighborhoods
S: Supplemental Material
SES: Socioeconomic status
