# Supplementary material for: The neighborhood social environment and physical activity: a systematic scoping review
Source: Int J Behav Nutr Phys Act. 2019 Dec 9;16:124. doi: 10.1186/s12966-019-0873-7 (PMC6902518; doi:10.1186/s12966-019-0873-7)
Supplement: Supplementary file 2 — Additional file 2. Full Search Terms. [file 12966_2019_873_MOESM2_ESM.docx]

| Environment | Physical Activity | Social Environment |
| --- | --- | --- |
| "local" or “neighborhood” or “neighborhoods”  or “neighbourhood” or “neighbourhoods”  or “community” or “environment” | “exercise” OR “exercising” OR “exerciser” OR “physical activity” OR “physically active” OR “play” OR “playing” OR "walk" or "walking" or "biking" or "bike" or "bicycling" or "bicycle" or "biker" | “social inequality” or “income inequality” or “racial discrimination” or “discrimination” or “institutional discrimination” or “racism” or “social cohesion” or “social connectedness” or “collective efficacy” or “social control” or “social capital” or “social composition” or “crime” or “deprivation” or “cultural norms” or “social norms” or “sociocultural” or “socio-cultural” or “civic participation” or “community participation” or “concentrated disadvantage” or "social environment" |
