# Supplementary material for: The neighborhood social environment and physical activity: a systematic scoping review
Source: Int J Behav Nutr Phys Act. 2019 Dec 9;16:124. doi: 10.1186/s12966-019-0873-7 (PMC6902518; doi:10.1186/s12966-019-0873-7)
Supplement: Supplementary file 3 — Additional file 3: Table S1. Overview of study characteristics. [file 12966_2019_873_MOESM3_ESM.docx]

| **Reference Number** | **First Author, Publication Year** | **Study Design** | **Study Location** | **Study Setting** | **Sample Size** | **% Male** | **Age** | **Objectively Measured PA (Yes/No)** |
| --- | --- | --- | --- | --- | --- | --- | --- | --- |
| (1) | Aarts, 2010 | Cross-sectional | Europe | Urban | 6,470 | 50.0 | Youth | No |
| (2) | Aarts, 2013 | Cross-sectional | Europe | Urban | 5963 | NR | Youth | No |
| (3) | Adams, 2009 | Cross-sectional | Australia/ New Zealand | Urban | 4060 | NR | Adult | No |
| (4) | Adlakha, 2017 | Cross-sectional | Asia | Urban | 370 | 53.8 | Adult | No |
| (5) | Adlakha , 2015 | Cross-sectional | North America | Urban | 1995 | 32.4 | Adult | No |
| (6) | Alton, 2007 | Cross-sectional | Europe | NR | 473 | 53.0 | Youth | No |
| (7) | Alves, 2013 | Cross-sectional | Europe | Urban | 2485 | 37.8 | Adult | No |
| (8) | Amorim, 2010 | Cross-sectional | South America | Urban | 972 | 43.0 | Adult | No |
| (9) | Andersen, 2015 | Cross-sectional | North America | Urban | 435 | 37.9 | Adult | No |
| (10) | Andrade, 2015 | Cross-sectional | South America | Urban | 3597 | 46.9 | Adult | No |
| (11) | Annear, 2009 | Cross-sectional | Australia/ New Zealand | Urban | 63 | 36.5 | Adult | No |
| (12) | Armstrong-Brown, 2014 | Cross-sectional | North America | Urban | 472 | 32.0 | Adult | No |
| (13) | Astell-Burt, 2015 | Cross-sectional | Australia/ New Zealand | NR | 203,883 | NR | Adult | No |
| (14) | Ball, 2010 | Cross-sectional | Australia/ New Zealand | Urban | 1405 | 0.0 | Adult | No |
| (15) | Beenackers, 2013 | Cross-sectional | Europe | NR | 4395 | 46.7 | Adult | No |
| (16) | Beets, 2008 | Cross-sectional | North America | NR | 10694 | 51.0 | Youth | No |
| (17) | Behanova, 2015 | Cross-sectional | Europe | Urban | 1404 | 53.0 | Adult | No |
| (18) | Boclin, 2014 | Longitudinal | South America | NR | 2674 | 42.5 | Adult | No |
| (19) | Bolivar, 2010 | Cross-sectional | Europe | NR | 13,193 | 49.0 | Both | No |
| (20) | Boone-Heinonen, 2011 | Cross-sectional | North America | Both | 4179 | 42.6 | Adult | No |
| (21) | Borrell, 2013 | Longitudinal | North America | NR | 2,491 | NR | Both | No |
| (22) | Bringolf-Isler, 2008 | Cross-sectional | Europe | Both | 1031 | 48.8 | Youth | No |
| (23) | Broyles , 2016 | Experimental | North America | Urban | 27 | 45.0 | Youth | Yes |
| (24) | Bungum, 2012 | Cross-sectional | North America | NR | 263 | 34.6 | Adult | No |
| (25) | Carlson, 2016 | Cross-sectional | North America | Both | 3973 | 49.1 | Adult | No |
| (26) | Carroll-Scott, 2013 | Cross-sectional | North America | Urban | 1048 | 47.6 | Youth | No |
| (27) | Carver, 2015 | Cross-sectional | Australia/ New Zealand | Both | 640 | 49.0 | Youth | No |
| (28) | Carver , 2010 | Cross-sectional | Australia/New Zealand | Both | 440 | 47.0 | Youth | Yes |
| (29) | Caspi, 2013 | Cross-sectional | North America | Urban | 828 | 20.0 | Adult | No |
| (30) | Cerin, 2016 | Cross-sectional | North America | Urban | 84 | 58.0 | Youth | Yes |
| (31) | Chaudhury, 2016 | Cross-sectional | North America | Urban | 434 | 35.6 | Adult | No |
| (32) | Chen, 2013 | Cross-sectional | Asia | Urban | 3806 | 52.4 | Adult | No |
| (33) | Cleland, 2008 | Longitudinal | Australia/New Zealand | Urban | 375 | 0.0 | Adult | No |
| (34) | Datar, 2015 | Cross-sectional | North America | Both | 903 | 48.0 | Youth | No |
| (35) | Datar , 2013 | Longitudinal | North America | Both | 18,900 | 51.2 | Youth | No |
| (36) | Davison, 2012 | Cross-sectional | North America | Rural | 767 | 51.1 | Both | No |
| (37) | Deweese, 2013 | Cross-sectional | North America | Urban | 901 | 50.0 | Youth | No |
| (38) | D'Haese, 2015 | Cross-sectional | Europe | Urban | 606 | 46.1 | Youth | Yes |
| (39) | Ding, 2013 | Cross-sectional | North America, South America, Europe, Asia, Australia/New Zealand | Urban | 11541 | 44.4 | Adult | No |
| (40) | Doyle, 2006 | Cross-sectional | North America | Urban | 9252 | 48.0 | Adult | No |
| (41) | Dragano, 2007 | Cross-sectional | Europe | Urban | 11554 | 46.7 | Adult | No |
| (42) | Duke, 2012 | Cross-sectional | North America | NR | 64,076 | 52.0 | Youth | No |
| (43) | Durand, 2012 | Cross-sectional | North America | Urban | 365 | 51.0 | Youth | Yes |
| (44) | Echeverria, 2008 | Cross-sectional | North America | Urban | 5943 | 48.0 | Adult | No |
| (45) | Echeverria, 2014 | Cross-sectional | North America | Urban | 107 | 45.0 | Youth | No |
| (46) | Edwards, 2013 | Cross-sectional | North America | Both | 280 | 30.0 | Adult | No |
| (47) | Eichinger, 2015 | Cross-sectional | Europe | Both | 904 | 57.9 | Adult | No |
| (48) | Esteban-Cornejo, 2016 | Cross-sectional | North America | Urban | 928 | 49.6 | Youth | No |
| (49) | Evenson, 2007 | Cross-sectional | North America | Urban | 1,554 | 0.0 | Youth | Yes |
| (50) | Evenson, 2012 | Cross-sectional | North America | Urban | 818 | 45.5 | Adult | No |
| (51) | Evenson , 2006 | Cross-sectional | North America | Urban | 610 | 0.0 | Youth | No |
| (52) | Forsyth, 2015 | Cross-sectional | North America | Urban | 2455 | 46.6 | Youth | No |
| (53) | Foster, 2014 | Cross-sectional | Australia/ New Zealand | Urban | 3,487 | 38.2 | Adult | No |
| (54) | Foster, 2016 | Longitudinal | Australia/ New Zealand | Urban | 531 | 40,3 | Adult | No |
| (55) | Fox, 2011 | Cross-sectional | Europe | Urban | 240 | 52.0 | Adult | Yes |
| (56) | Gallagher, 2014 | Cross-sectional | North America | Urban | 362 | 29.0 | Adult | No |
| (57) | Gao, 2015 | Cross-sectional | Asia | Urban | 2,783 | 41.0 | Adult | No |
| (58) | Garrett, 2012 | Cross-sectional | Australia/New Zealand | Both | 8038 | 40.0 | Both | No |
| (59) | Gay, 2011 | Cross-sectional | North America | Urban | 477 | 13.8 | Adult | No |
| (60) | Glass , 2006 | Cross-sectional | North America | Urban | 1140 | 34.0 | Adult | No |
| (61) | Gomes, 2016 | Cross-sectional | South America | Urban | 5779 | 41.2 | Adult | No |
| (62) | Graziose, 2016 | Cross-sectional | North America | Urban | 952 | 49.0 | Youth | No |
| (63) | Halbert, 2014 | Cross-sectional | North America | Urban | 338 | 43.0 | Adult | No |
| (64) | Handy, 2008 | Cross-sectional/Longitudinal | North America | Urban | 1682 | 52.5 | Adult | No |
| (65) | Harrison, 2007 | Cross-sectional | Europe | NR | 15,461 | 45.0 | Adult | No |
| (66) | Heitzler, 2006 | Cross-sectional | North America | NR | 3114 | 51.1 | Youth | No |
| (67) | Hume, 2007 | Cross-sectional | Australia/New Zealand | Urban | 280 | 49.0 | Youth | Yes |
| (68) | Inoue, 2011 | Cross-sectional | Asia | Both | 1921 | 50.9 | Adult | No |
| (69) | Jack, 2014 | Cross-sectional | North America | NR | 1875 | 38.0 | Adult | No |
| (70) | Jago, 2006 | Cross-sectional | North America | Urban | 210 | 100.0 | Youth | Yes |
| (71) | Jago, 2006 | Cross-sectional | North America | NR | 210 | 100.0 | Youth | Yes |
| (72) | Janke, 2016 | Cross-sectional | Europe | Both | 964,318 | 41.0 | Both | No |
| (73) | Jilcott, 2015 | Cross-sectional | North America | Rural | 366 | 24.0 | Adult | Yes |
| (74) | Jones, 2009 | Cross-sectional | Europe | Urban | 6,821 | 41.0 | NR | No |
| (75) | Kaczynski, 2012 | Cross-sectional | North America | Urban | 380 | 36.0 | Adult | No |
| (76) | Kamphuis, 2008 | Cross-sectional | Europe | NR | 4785 | 47.5 | Adult | No |
| (77) | Karusisi, 2012 | Cross-sectional | Europe | Urban | 7290 | NR | Adult | No |
| (78) | Katapally, 2015 | Cross-sectional | North America | Urban | 331 | 50.0 | Youth | Yes |
| (79) | Kelly, 2013 | Cross-sectional | North America | Urban | NR | 47.1 | Adult | No |
| (80) | Kerr, 2008 | Cross-sectional | North America | Urban | 878 | 46.0 | Youth | No |
| (81) | Kerr, 2006 | Cross-sectional | North America | Urban | 259 | 51.4 | Youth | No |
| (82) | Kerr, 2015 | Longitudinal | North America | Urban | 796 | 54.9 | Adult | No |
| (83) | Kim, 2016 | Cross-sectional | North America | Both | 842 | NR | Youth | No |
| (84) | Kimbro, 2011 | Cross-sectional | North America | Urban | 1822 | 51.0 | Youth | No |
| (85) | King, 2008 | Cross-sectional | North America | NR | 190 | 43.0 | Adult | No |
| (86) | Kneeshaw-Price, 2015 | Cross-sectional | North America | Urban | 145 | 49.0 | Youth | Yes |
| (87) | Kramer, 2013 | Cross-sectional | Europe | NR | 20046 | 47.2 | Youth | No |
| (88) | Kramer, 2015 | Cross-sectional | Europe | NR | 20,600 | 47.8 | Adult | No |
| (89) | Kremers, 2012 | Cross-sectional | Europe | Urban | 470 | 48.9 | Adult | No |
| (90) | Kuo , 2007 | Cross-sectional | North America | Urban | 221 | 0.0 | Youth | No |
| (91) | Kurka, 2015 | Cross-sectional | North America | Urban | 678 | 51.0 | Youth | Yes |
| (92) | Lavin Fueyo, 2016 | Cross-sectional | South America | Urban | 1777 | 53.0 | Youth | No |
| (93) | Lee, 2007 | Cross-sectional | North America | Urban | 2672 | 0.0 | Both | No |
| (94) | Leslie, 2010 | Cross-sectional | Australia/New Zealand | NR | 502 | 36.0 | Adult | No |
| (95) | Li, 2006 | Cross-sectional | Asia | Urban | 1787 | 50.0 | Youth | No |
| (96) | Li, 2015 | Cross-sectional | North America | NR | 1045 | NR | Adult | No |
| (97) | Liao, 2011 | Cross-sectional | Asia | NR | 1420 | 100.0 | Adult | No |
| (98) | Loch, 2015 | Cross-sectional | South America | NR | 1062 | 50.0 | Adult | No |
| (99) | Logstein, 2013 | Cross-sectional | Europe | NR | 8114 | 50.0 | Youth | No |
| (100) | Lovasi, 2011 | Cross-sectional | North America | Urban | 428 | 50.0 | Youth | Yes |
| (101) | Machado-Rodrigues, 2014 | Cross-sectional | Europe | NR | 1186 | 0.0 | Youth | No |
| (102) | Mackenbach, 2016 | Cross-sectional | Europe | Urban | 5205 | 45.0 | Adult | No |
| (103) | Mackenbach, 2016 | Cross-sectional | Europe | Urban | 5900 | 44.1 | Adult | No |
| (104) | Macniven, 2016 | Cross-sectional | Australia/New Zealand | NR | 59489 | 43.4 | Adult | No |
| (105) | Maisel, 2016 | Cross-sectional | North America | Both | 121 | 26.5 | Adult | No |
| (106) | Marlier, 2015 | Cross-sectional | Europe | NR | 414 | 45.6 | Adult | No |
| (107) | Mason, 2013 | Cross-sectional | Europe | Urban | 3,824 | 43.0 | Both | No |
| (108) | McDonald, 2007 | Cross-sectional | North America | Urban | 614 | 49.0 | Youth | No |
| (109) | McDonald, 2008 | Cross-sectional | North America | NR | 359 | 48.0 | Adult | No |
| (110) | McGinn, 2008 | Cross-sectional | North America | NR | 1961 | 33.0 | Adult | No |
| (111) | Mendes, 2009 | Cross-sectional | North America | Urban | 4,317 | 39.0 | Adult | No |
| (112) | Merom, 2009 | Experimental | Australia/New Zealand | NR | 314 | 15.0 | Adult | Yes |
| (113) | Mohnen, 2012 | Cross-sectional | Europe | NR | 9,253 | 44.5 | Adult | No |
| (114) | Mota, 2007 | Cross-sectional | Europe | Urban | 181 | 30.4 | Adult | No |
| (115) | Mota, 2009 | Cross-sectional | Europe | Urban | 162 | 0.0 | Youth | No |
| (116) | Mota, 2009 | Cross-sectional | Europe | NR | 425 | 0.0 | Youth | No |
| (117) | Motl, 2007 | Cross-sectional | North America | NR | 1655 | 0.0 | Youth | No |
| (118) | Muthuri, 2016 | Cross-sectional | Africa | Urban | 563 | 46.5 | Youth | Yes |
| (119) | Nehme, 2016 | Cross-sectional | North America | Urban | 231 | 35.2 | Adult | No |
| (120) | Noonan, 2016 | Cross-sectional | Europe | NR | 194 | 45.0 | Youth | No |
| (121) | Oh, 2010 | Cross-sectional | North America | Urban | 281 | 0.0 | Adult | Yes |
| (122) | Oluyomi, 2014 | Cross-sectional | North America | NR | 830 | 49.6 | Youth | No |
| (123) | Osypuk, 2009 | Cross-sectional | North America | Urban | 1902 | 49.0 | Adult | No |
| (124) | Oyeyemi, 2012 | Cross-sectional | Africa | Urban | 219 | 60.7 | Adult | Yes |
| (125) | Oyeyemi, 2015 | Cross-sectional | Africa | Urban | 613 | 68.0 | Adult | No |
| (126) | Pabayo, 2014 | Cross-sectional | North America | Urban | 1878 | 44.0 | Both | No |
| (127) | Peachey, 2015 | Cross-sectional | North America | Urban | 822 | 29.1 | Both | No |
| (128) | Pelclova, 2014 | Cross-sectional | Europe | NR | 2,839 | 50.0 | Adult | No |
| (129) | Perez, 2017 | Cross-sectional | North America | Urban | 436 | 0.0 | Adult | Yes |
| (130) | Piro, 2006 | Cross-sectional | Europe | NR | 3499 | 43.0 | Adult | No |
| (131) | Quon, 2014 | Cross-sectional | North America | NR | 2199 | 48.0 | Youth | No |
| (132) | Rech, 2014 | Cross-sectional | South America | Urban | 1461 | 36.3 | Adult | No |
| (133) | Reis, 2014 | Cross-sectional | South America | Urban | 6166 | 37.1 | Adult | No |
| (134) | Remmers, 2014 | Longitudinal | Europe | NR | 1407 | 51.0 | Youth | No |
| (135) | Rhodes, 2006 | Cross-sectional | North America | NR | 351 | 50.4 | Adult | No |
| (136) | Ribeiro, 2015 | Cross-sectional | Europe | Urban | 532 | 39.0 | Adult | No |
| (137) | Rind, 2015 | Cross-sectional | Europe | Urban | 20146 | NR | Both | No |
| (138) | Robinson, 2016 | Cross-sectional | North America | Urban | 80 | 44.0 | Youth | Yes |
| (139) | Rossen, 2011 | Cross-sectional | North America | Urban | 365 | 46.0 | Youth | No |
| (140) | Salahuddin, 2016 | Cross-sectional | North America | Both | 835 | 49.2 | Youth | No |
| (141) | Sallis, 2007 | Experimental | North America | NR | 861 | 55.1 | Adult | No |
| (142) | Salmon, 2013 | Cross-sectional | North America | Urban | 613 | 47.0 | Both | Yes |
| (143) | Samuel, 2015 | Cross-sectional | North America | Urban | 5381 | 47.2 | Adult | No |
| (144) | Santana, 2009 | Cross-sectional | Europe | Urban | 7669 | 46.5 | Adult | No |
| (145) | Santos, 2008 | Cross-sectional | Europe | Both | 7330 | 44.0 | Adult | No |
| (146) | Santos, 2009 | Cross-sectional | Europe | Both | 7330 | 44.0 | Adult | No |
| (147) | Schoeny, 2017 | Experimental | North America | Urban | 288 | 0.0 | Adult | Yes |
| (148) | Schulz, 2013 | Cross-sectional | North America | Urban | 919 | 47.9 | Adult | No |
| (149) | Shenassa, 2006 | Cross-sectional | Europe | Both | 5338 | 46.1 | Adult | No |
| (150) | Siceloff, 2014 | Cross-sectional | North America | NR | 434 | 37.0 | Adult | Yes |
| (151) | Singh, 2008 | Cross-sectional | North America | Both | 68,288 | 51,50 | Youth | No |
| (152) | Singh, 2009 | Cross-sectional | North America | Both | 68,288 | 52.0 | Youth | No |
| (153) | Solomon, 2013 | Cross-sectional | Europe | Rural | 2415 | 37.3 | Adult | No |
| (154) | Soltero, 2015 | Cross-sectional | North America | Urban | 410 | 0.0 | Adult | No |
| (155) | Stimpson, 2007 | Cross-sectional | North America | NR | 20,050 | 50.0 | Both | No |
| (156) | Strath, 2012 | Cross-sectional | North America | NR | 148 | 25.0 | Adult | Yes |
| (157) | Strong, 2013 | Cross-sectional | North America | Urban | 1374 | 25.4 | Youth | No |
| (158) | Sugiyama, 2015 | Cross-sectional | Australia/ New Zealand | NR | 1653 | 45.0 | Adult | No |
| (159) | Sugiyama, 2015 | Longitudinal | Australia/ New Zealand | NR | 2684 | 44.0 | Adult | No |
| (160) | Tanaka, 2016 | Cross-sectional | Asia | Rural | 730 | 43.0 | Adult | No |
| (161) | Tappe, 2013 | Cross-sectional | North America | Urban | 724 | 50.7 | Youth | Yes |
| (162) | Timperio, 2006 | Cross-sectional | Australia/New Zealand | Urban | 912 | 48.7 | Youth | No |
| (163) | Timperio, 2015 | Cross-sectional | Australia/New Zealand | Urban | 2784 | 0.0 | Adult | No |
| (164) | Troped, 2011 | Cross-sectional | North America | NR | 68968 | 0.0 | Adult | No |
| (165) | Turrell, 2010 | Longitudinal | Australia/ New Zealand | NR | 11,037 | 44.1 | Adult | No |
| (166) | Ueshima, 2010 | Cross-sectional | Asia | Urban | 2260 | 39.2 | Adult | No |
| (167) | Vamos, 2015 | Cross-sectional | North America | NR | 7,596 | 0.0 | Adult | No |
| (168) | Van Cauwenberg, 2014 | Cross-sectional | Europe | Both | 50,986 | 54.0 | Adult | No |
| (169) | Van Dyck, 2015 | Cross-sectional | North America, South America, Europe, Asia, Australia/New Zealand | Urban | 7273 | 46.0 | Adult | Yes |
| (170) | Van Holle, 2016 | Cross-sectional | Europe | Urban | 438 | 45.9 | Adult | Yes |
| (171) | van Loon, 2014 | Cross-sectional | North America | NR | 366 | 47.5 | Youth | Yes |
| (172) | Vanwolleghem, 2016 | Longitudinal | Europe | NR | 313 | 53.8 | Youth | No |
| (173) | Verhoeven, 2016 | Cross-sectional | Europe | Urban | 562 | 45.4 | Youth | No |
| (174) | Voorhees, 2009 | Cross-sectional | North America | Urban | 1603 | 0.0 | Adult | Yes |
| (175) | Wang, 2010 | Cross-sectional | North America | NR | 114 | 18.4 | Adult | No |
| (176) | Weber Corseuil, 2012 | Cross-sectional | South America | Urban | 1,656 | 37.0 | Adult | No |
| (177) | Weir, 2006 | Cross-sectional | North America | Urban | 307 | 51.5 | Youth | No |
| (178) | Wen, 2007 | Cross-sectional | North America | NR | 41,545 | 49.0 | Adult | No |
| (179) | Wen, 2009 | Cross-sectional | North America | Urban | 4437 | 38.5 | Adult | No |
| (180) | Zandieh, 2016 | Cross-sectional | Europe | Urban | 173 | 43.0 | Adult | Yes |
| (181) | Zhu, 2008 | Cross-sectional | North America | Urban | 1281 | NR | Youth | No |

1. Aarts MJ, Wendel-Vos W, van Oers HA, van de Goor IA, Schuit AJ. Environmental determinants of outdoor play in children: a large-scale cross-sectional study. Am J Prev Med. 2010;39(3):212-9.

2. Aarts MJ, Mathijssen JJ, van Oers JA, Schuit AJ. Associations between environmental characteristics and active commuting to school among children: a cross-sectional study. International journal of behavioral medicine. 2013;20(4):538-55.

3. Adams RJ, Howard N, Tucker G, Appleton S, Taylor AW, Chittleborough C, et al. Effects of area deprivation on health risks and outcomes: a multilevel, cross-sectional, Australian population study. International journal of public health. 2009;54(3):183-92.

4. Adlakha D, Hipp JA, Brownson RC, A AE, C KL, Raghavan R. "Can we walk?" Environmental supports for physical activity in India. Prev Med. 2017;103s:S81-s9.

5. Adlakha D, Hipp AJ, Marx C, Yang L, Tabak R, Dodson EA, et al. Home and workplace built environment supports for physical activity. Am J Prev Med. 2015;48(1):104-7.

6. Alton D, Adab P, Roberts L, Barrett T. Relationship between walking levels and perceptions of the local neighbourhood environment. Arch Dis Child. 2007;92(1):29-33.

7. Alves L, Silva S, Severo M, Costa D, Pina MF, Barros H, et al. Association between neighborhood deprivation and fruits and vegetables consumption and leisure-time physical activity: a cross-sectional multilevel analysis. BMC Public Health. 2013;13:1103.

8. Amorim TC, Azevedo MR, Hallal PC. Physical activity levels according to physical and social environmental factors in a sample of adults living in South Brazil. J Phys Act Health. 2010;7 Suppl 2:S204-12.

9. Andersen L, Gustat J, Becker AB. The Relationship Between the Social Environment and Lifestyle-Related Physical Activity in a Low-Income African American Inner-City Southern Neighborhood. J Commun Health. 2015;40(5):967-74.

10. Andrade AC, Peixoto SV, Friche AA, Goston JL, Cesar CC, Xavier CC, et al. Social context of neighborhood and socioeconomic status on leisure-time physical activity in a Brazilian urban center: The BH Health Study. Cadernos de saude publica. 2015;31 Suppl 1:136-47.

11. Annear MJ, Cushman G, Gidlow B. Leisure time physical activity differences among older adults from diverse socioeconomic neighborhoods. Health & place. 2009;15(2):482-90.

12. Armstrong-Brown J, Eng E, Hammond WP, Zimmer C, Bowling JM. Redefining Racial Residential Segregation and its Association With Physical Activity Among African Americans 50 Years and Older: A Mixed Methods Approach. Journal of aging and physical activity. 2014.

13. Astell-Burt T, Feng X, Kolt GS. Identification of the impact of crime on physical activity depends upon neighbourhood scale: Multilevel evidence from 203,883 Australians. Health & place. 2015;31:120-3.

14. Ball K, Cleland VJ, Timperio AF, Salmon J, Giles-Corti B, Crawford DA. Love thy neighbour? Associations of social capital and crime with physical activity amongst women. Soc Sci Med. 2010;71(4):807-14.

15. Beenackers MA, Kamphuis CB, Mackenbach JP, Burdorf A, van Lenthe FJ. Why some walk and others don't: exploring interactions of perceived safety and social neighborhood factors with psychosocial cognitions. Health education research. 2013;28(2):220-33.

16. Beets MW, Foley JT. Association of father involvement and neighborhood quality with kindergartners' physical activity: a multilevel structural equation model. Am J Health Promot. 2008;22(3):195-203.

17. Behanova M, Katreniakova Z, Nagyova I, van Ameijden EJ, Dijkshoorn H, van Dijk JP, et al. The effect of neighbourhood unemployment on health-risk behaviours in elderly differs between Slovak and Dutch cities. European journal of public health. 2015;25(1):108-14.

18. Boclin Kde L, Faerstein E, Leon AC. Neighborhood contextual characteristics and leisure-time physical activity: Pro-Saude Study. Revista de saude publica. 2014;48(2):249-57.

19. Bolivar J, Daponte A, Rodriguez M, Sanchez JJ. The influence of individual, social and physical environment factors on physical activity in the adult population in Andalusia, Spain. Int J Environ Res Public Health. 2010;7(1):60-77.

20. Boone-Heinonen J, Gordon-Larsen P. Life stage and sex specificity in relationships between the built and socioeconomic environments and physical activity. Journal of epidemiology and community health. 2011;65(10):847-52.

21. Borrell LN, Kiefe CI, Diez-Roux AV, Williams DR, Gordon-Larsen P. Racial discrimination, racial/ethnic segregation, and health behaviors in the CARDIA study. Ethn Health. 2013;18(3):227-43.

22. Bringolf-Isler B, Grize L, Mader U, Ruch N, Sennhauser FH, Braun-Fahrlander C. Personal and environmental factors associated with active commuting to school in Switzerland. Prev Med. 2008;46(1):67-73.

23. Broyles ST, Myers CA, Drazba KT, Marker AM, Church TS, Newton RL, Jr. The Influence of Neighborhood Crime on Increases in Physical Activity during a Pilot Physical Activity Intervention in Children. J Urban Health. 2016;93(2):271-8.

24. Bungum TJ, Landers M, Azzarelli M, Moonie S. Perceived environmental physical activity correlates among Asian Pacific Islander Americans. J Phys Act Health. 2012;9(8):1098-104.

25. Carlson SA, Paul P, Watson KB, Schmid TL, Fulton JE. How reported usefulness modifies the association between neighborhood supports and walking behavior. Prev Med. 2016;91:76-81.

26. Carroll-Scott A, Gilstad-Hayden K, Rosenthal L, Peters SM, McCaslin C, Joyce R, et al. Disentangling neighborhood contextual associations with child body mass index, diet, and physical activity: the role of built, socioeconomic, and social environments. Soc Sci Med. 2013;95:106-14.

27. Carver A, Timperio AF, Crawford DA. Bicycles gathering dust rather than raising dust--Prevalence and predictors of cycling among Australian schoolchildren. Journal of science and medicine in sport. 2015;18(5):540-4.

28. Carver A, Timperio A, Hesketh K, Crawford D. Are children and adolescents less active if parents restrict their physical activity and active transport due to perceived risk? Soc Sci Med. 2010;70(11):1799-805.

29. Caspi CE, Kawachi I, Subramanian SV, Tucker-Seeley R, Sorensen G. The social environment and walking behavior among low-income housing residents. Soc Sci Med. 2013;80:76-84.

30. Cerin E, Baranowski T, Barnett A, Butte N, Hughes S, Lee RE, et al. Places where preschoolers are (in)active: an observational study on Latino preschoolers and their parents using objective measures. The international journal of behavioral nutrition and physical activity. 2016;13:29.

31. Chaudhury H, Campo M, Michael Y, Mahmood A. Neighbourhood environment and physical activity in older adults. Soc Sci Med. 2016;149:104-13.

32. Chen TA, Lee JS, Kawakubo K, Watanabe E, Mori K, Kitaike T, et al. Features of perceived neighborhood environment associated with daily walking time or habitual exercise: differences across gender, age, and employment status in a community-dwelling population of Japan. Environmental health and preventive medicine. 2013;18(5):368-76.

33. Cleland VJ, Timperio A, Crawford D. Are perceptions of the physical and social environment associated with mothers' walking for leisure and for transport? A longitudinal study. Prev Med. 2008;47(2):188-93.

34. Datar A, Nicosia N, Wong E, Shier V. Neighborhood Environment and Children's Physical Activity and Body Mass Index: Evidence from Military Personnel Installation Assignments. Childhood obesity (Print). 2015.

35. Datar A, Nicosia N, Shier V. Parent perceptions of neighborhood safety and children's physical activity, sedentary behavior, and obesity: evidence from a national longitudinal study. Am J Epidemiol. 2013;177(10):1065-73.

36. Davison KK, Nishi A, Kranz S, Wyckoff L, May JJ, Earle-Richardson GB, et al. Associations among social capital, parenting for active lifestyles, and youth physical activity in rural families living in upstate New York. Soc Sci Med. 2012;75(8):1488-96.

37. Deweese RS, Yedidia MJ, Tulloch DL, Ohri-Vachaspati P. Neighborhood perceptions and active school commuting in low-income cities. Am J Prev Med. 2013;45(4):393-400.

38. D'Haese S, Van Dyck D, De Bourdeaudhuij I, Deforche B, Cardon G. The association between the parental perception of the physical neighborhood environment and children's location-specific physical activity. BMC Public Health. 2015;15:565.

39. Ding D, Adams MA, Sallis JF, Norman GJ, Hovell MF, Chambers CD, et al. Perceived neighborhood environment and physical activity in 11 countries: do associations differ by country? The international journal of behavioral nutrition and physical activity. 2013;10:57.

40. Doyle S, Kelly-Schwartz A, Schlossberg M, Stockard J. Active Community Environments and Health: The Relationship of Walkable and Safe Communities to Individual Health. Journal of the American Planning Association. 2006;72(1):19-31.

41. Dragano N, Bobak M, Wege N, Peasey A, Verde PE, Kubinova R, et al. Neighbourhood socioeconomic status and cardiovascular risk factors: a multilevel analysis of nine cities in the Czech Republic and Germany. BMC Public Health. 2007;7:255.

42. Duke NN, Borowsky IW, Pettingell SL. Parent perceptions of neighborhood: relationships with US youth physical activity and weight status. Maternal and child health journal. 2012;16(1):149-57.

43. Durand CP, Dunton GF, Spruijt-Metz D, Pentz MA. Does community type moderate the relationship between parent perceptions of the neighborhood and physical activity in children? Am J Health Promot. 2012;26(6):371-80.

44. Echeverria S, Diez-Roux AV, Shea S, Borrell LN, Jackson S. Associations of neighborhood problems and neighborhood social cohesion with mental health and health behaviors: the Multi-Ethnic Study of Atherosclerosis. Health & place. 2008;14(4):853-65.

45. Echeverria SE, Luan Kang A, Isasi CR, Johnson-Dias J, Pacquiao D. A community survey on neighborhood violence, park use, and physical activity among urban youth. J Phys Act Health. 2014;11(1):186-94.

46. Edwards M, Cunningham G. Examining the associations of perceived community racism with self-reported physical activity levels and health among older racial minority adults. J Phys Act Health. 2013;10(7):932-9.

47. Eichinger M, Titze S, Haditsch B, Dorner TE, Stronegger WJ. How are physical activity behaviors and cardiovascular risk factors associated with characteristics of the built and social residential environment? PLoS One. 2015;10(6):e0126010.

48. Esteban-Cornejo I, Carlson JA, Conway TL, Cain KL, Saelens BE, Frank LD, et al. Parental and Adolescent Perceptions of Neighborhood Safety Related to Adolescents' Physical Activity in Their Neighborhood. Research quarterly for exercise and sport. 2016;87(2):191-9.

49. Evenson KR, Scott MM, Cohen DA, Voorhees CC. Girls' perception of neighborhood factors on physical activity, sedentary behavior, and BMI. Obesity (Silver Spring, Md). 2007;15(2):430-45.

50. Evenson KR, Block R, Diez Roux AV, McGinn AP, Wen F, Rodriguez DA. Associations of adult physical activity with perceived safety and police-recorded crime: the Multi-ethnic Study of Atherosclerosis. The international journal of behavioral nutrition and physical activity. 2012;9:146.

51. Evenson KR, Birnbaum AS, Bedimo-Rung AL, Sallis JF, Voorhees CC, Ring K, et al. Girls' perception of physical environmental factors and transportation: reliability and association with physical activity and active transport to school. The international journal of behavioral nutrition and physical activity. 2006;3:28.

52. Forsyth A, Wall M, Choo T, Larson N, Van Riper D, Neumark-Sztainer D. Perceived and Police-Reported Neighborhood Crime: Linkages to Adolescent Activity Behaviors and Weight Status. The Journal of adolescent health : official publication of the Society for Adolescent Medicine. 2015;57(2):222-8.

53. Foster S, Knuiman M, Villanueva K, Wood L, Christian H, Giles-Corti B. Does walkable neighbourhood design influence the association between objective crime and walking? The international journal of behavioral nutrition and physical activity. 2014;11:100.

54. Foster S, Hooper P, Knuiman M, Christian H, Bull F, Giles-Corti B. Safe RESIDential Environments? A longitudinal analysis of the influence of crime-related safety on walking. The international journal of behavioral nutrition and physical activity. 2016;13:22.

55. Fox KR, Hillsdon M, Sharp D, Cooper AR, Coulson JC, Davis M, et al. Neighbourhood deprivation and physical activity in UK older adults. Health & place. 2011;17(2):633-40.

56. Gallagher NA, Clarke PJ, Gretebeck KA. Gender differences in neighborhood walking in older adults. J Aging Health. 2014;26(8):1280-300.

57. Gao J. Association between social and built environments and leisure-time physical activity among Chinese older adults--a multilevel analysis. BMC Public Health. 2015;15:1317-28.

58. Garrett N, Schluter PJ, Schofield G. Physical activity profiles and perceived environmental determinants in New Zealand: a national cross-sectional study. J Phys Act Health. 2012;9(3):367-77.

59. Gay JL, Saunders RP, Dowda M. The relationship of physical activity and the built environment within the context of self-determination theory. Annals of behavioral medicine : a publication of the Society of Behavioral Medicine. 2011;42(2):188-96.

60. Glass TA, Rasmussen MD, Schwartz BS. Neighborhoods and obesity in older adults: the Baltimore Memory Study. Am J Prev Med. 2006;31(6):455-63.

61. Gomes CS, Matozinhos FP, Mendes LL, Pessoa MC, Velasquez-Melendez G. Physical and Social Environment Are Associated to Leisure Time Physical Activity in Adults of a Brazilian City: A Cross-Sectional Study. PLoS One. 2016;11(2):e0150017.

62. Graziose MM, Gray HL, Quinn J, Rundle AG, Contento IR, Koch PA. Association Between the Built Environment in School Neighborhoods With Physical Activity Among New York City Children, 2012. Preventing chronic disease. 2016;13:E110.

63. Halbert CH, Bellamy S, Briggs V, Bowman M, Delmoor E, Kumanyika S, et al. Collective efficacy and obesity-related health behaviors in a community sample of African Americans. J Community Health. 2014;39(1):124-31.

64. Handy SL, Cao X, Mokhtarian PL. The causal influence of neighborhood design on physical activity within the neighborhood: evidence from Northern California. Am J Health Promot. 2008;22(5):350-8.

65. Harrison RA, Gemmell I, Heller RF. The population effect of crime and neighbourhood on physical activity: an analysis of 15,461 adults. Journal of epidemiology and community health. 2007;61(1):34-9.

66. Heitzler CD, Martin SL, Duke J, Huhman M. Correlates of physical activity in a national sample of children aged 9-13 years. Prev Med. 2006;42(4):254-60.

67. Hume C, Salmon J, Ball K. Associations of children's perceived neighborhood environments with walking and physical activity. Am J Health Promot. 2007;21(3):201-7.

68. Inoue S, Ohya Y, Odagiri Y, Takamiya T, Kamada M, Okada S, et al. Perceived neighborhood environment and walking for specific purposes among elderly Japanese. Journal of epidemiology / Japan Epidemiological Association. 2011;21(6):481-90.

69. Jack E, McCormack GR. The associations between objectively-determined and self-reported urban form characteristics and neighborhood-based walking in adults. The international journal of behavioral nutrition and physical activity. 2014;11:71.

70. Jago R, Baranowski T, Baranowski JC. Observed, GIS, and self-reported environmental features and adolescent physical activity. Am J Health Promot. 2006;20(6):422-8.

71. Jago R, Baranowski T, Harris M. Relationships Between GIS Environmental Features and Adolescent Male Physical Activity: GIS Coding Differences. J Phys Act Health. 2006;3(2):230-42.

72. Janke K, Propper C, Shields MA. Assaults, murders and walkers: The impact of violent crime on physical activity. Journal of health economics. 2016;47:34-49.

73. Jilcott Pitts SB, Keyserling TC, Johnston LF, Smith TW, McGuirt JT, Evenson KR, et al. Associations between neighborhood-level factors related to a healthful lifestyle and dietary intake, physical activity, and support for obesity prevention polices among rural adults. J Community Health. 2015;40(2):276-84.

74. Jones A, Hillsdon M, Coombes E. Greenspace access, use, and physical activity: understanding the effects of area deprivation. Prev Med. 2009;49(6):500-5.

75. Kaczynski AT, Glover TD. Talking the talk, walking the walk: examining the effect of neighbourhood walkability and social connectedness on physical activity. J Public Health (Oxf). 2012;34(3):382-9.

76. Kamphuis CB, Van Lenthe FJ, Giskes K, Huisman M, Brug J, Mackenbach JP. Socioeconomic status, environmental and individual factors, and sports participation. Medicine and science in sports and exercise. 2008;40(1):71-81.

77. Karusisi N, Bean K, Oppert JM, Pannier B, Chaix B. Multiple dimensions of residential environments, neighborhood experiences, and jogging behavior in the RECORD Study. Prev Med. 2012;55(1):50-5.

78. Katapally TR, Muhajarine N. Capturing the Interrelationship between Objectively Measured Physical Activity and Sedentary Behaviour in Children in the Context of Diverse Environmental Exposures. Int J Environ Res Public Health. 2015;12(9):10995-1011.

79. Kelly CM, Lian M, Struthers J, Kammrath A. Walking to Work: The Roles of Neighborhood Walkability and Socioeconomic Deprivation. J Phys Act Health. 2013.

80. Kerr J, Norman GJ, Sallis JF, Patrick K. Exercise aids, neighborhood safety, and physical activity in adolescents and parents. Medicine and science in sports and exercise. 2008;40(7):1244-8.

81. Kerr J, Rosenberg D, Sallis JF, Saelens BE, Frank LD, Conway TL. Active commuting to school: Associations with environment and parental concerns. Medicine and science in sports and exercise. 2006;38(4):787-94.

82. Kerr Z, Evenson KR, Moore K, Block R, Diez Roux AV. Changes in walking associated with perceived neighborhood safety and police-recorded crime: The multi-ethnic study of atherosclerosis. Prev Med. 2015;73:88-93.

83. Kim HJ, Heinrich KM. Built Environment Factors Influencing Walking to School Behaviors: A Comparison between a Small and Large US City. Frontiers in public health. 2016;4:77.

84. Kimbro RT, Brooks-Gunn J, McLanahan S. Young children in urban areas: links among neighborhood characteristics, weight status, outdoor play, and television watching. Soc Sci Med. 2011;72(5):668-76.

85. King D. Neighborhood and individual factors in activity in older adults: results from the neighborhood and senior health study. Journal of aging and physical activity. 2008;16(2):144-70.

86. Kneeshaw-Price SH, Saelens BE, Sallis JF, Frank LD, Grembowski DE, Hannon PA, et al. Neighborhood Crime-Related Safety and Its Relation to Children's Physical Activity. J Urban Health. 2015;92(3):472-89.

87. Kramer D, Maas J, Wingen M, Kunst AE. Neighbourhood safety and leisure-time physical activity among Dutch adults: a multilevel perspective. The international journal of behavioral nutrition and physical activity. 2013;10:11.

88. Kramer D, Stronks K, Maas J, Wingen M, Kunst AE. Social neighborhood environment and sports participation among Dutch adults: does sports location matter? Scandinavian journal of medicine & science in sports. 2015;25(2):273-9.

89. Kremers SP, de Bruijn GJ, Visscher TL, Deeg DJ, Thomese GC, Visser M, et al. Associations between safety from crime, cycling, and obesity in a Dutch elderly population: results from the Longitudinal Aging Study Amsterdam. Journal of environmental and public health. 2012;2012:127857.

90. Kuo J, Voorhees CC, Haythornthwaite JA, Young DR. Associations between family support, family intimacy, and neighborhood violence and physical activity in urban adolescent girls. Am J Public Health. 2007;97(1):101-3.

91. Kurka JM, Adams MA, Todd M, Colburn T, Sallis JF, Cain KL, et al. Patterns of neighborhood environment attributes in relation to children's physical activity. Health & place. 2015;34:164-70.

92. Lavin Fueyo J, Totaro Garcia LM, Mamondi V, Pereira Alencar G, Florindo AA, Berra S. Neighborhood and family perceived environments associated with children's physical activity and body mass index. Prev Med. 2016;82:35-41.

93. Lee RE, Cubbin C, Winkleby M. Contribution of neighbourhood socioeconomic status and physical activity resources to physical activity among women. Journal of epidemiology and community health. 2007;61(10):882-90.

94. Leslie E, Cerin E, Kremer P. Perceived neighborhood environment and park use as mediators of the effect of area socio-economic status on walking behaviors. J Phys Act Health. 2010;7(6):802-10.

95. Li M, Dibley MJ, Sibbritt D, Yan H. Factors associated with adolescents' physical inactivity in Xi'an City, China. Medicine and science in sports and exercise. 2006;38(12):2075-85.

96. Li Y, Kao D, Dinh TQ. Correlates of neighborhood environment with walking among older Asian Americans. J Aging Health. 2015;27(1):17-34.

97. Liao Y, Harada K, Shibata A, Ishii K, Oka K, Nakamura Y, et al. Perceived environmental factors associated with physical activity among normal-weight and overweight Japanese men. Int J Environ Res Public Health. 2011;8(4):931-43.

98. Loch MR, Souza RKTd, Mesas AE, Martinez-Gómez D, Rodríguez-Artalejo F. Relationship between social capital indicators and lifestyle in Brazilian adults. Cadernos de Saúde Pública. 2015;31:1636-47.

99. Logstein B, Blekesaune A, Almas R. Physical activity among Norwegian adolescents--a multilevel analysis of how place of residence is associated with health behaviour: the Young-HUNT study. International journal for equity in health. 2013;12:56.

100. Lovasi GS, Jacobson JS, Quinn JW, Neckerman KM, Ashby-Thompson MN, Rundle A. Is the environment near home and school associated with physical activity and adiposity of urban preschool children? J Urban Health. 2011;88(6):1143-57.

101. Machado-Rodrigues AM, Santana A, Gama A, Mourao I, Nogueira H, Rosado V, et al. Parental perceptions of neighborhood environments, BMI, and active behaviors in girls aged 7-9 years. American journal of human biology : the official journal of the Human Biology Council. 2014;26(5):670-5.

102. Mackenbach JD, Lakerveld J, van Lenthe FJ, Bardos H, Glonti K, Compernolle S, et al. Exploring why residents of socioeconomically deprived neighbourhoods have less favourable perceptions of their neighbourhood environment than residents of wealthy neighbourhoods. Obesity reviews : an official journal of the International Association for the Study of Obesity. 2016;17 Suppl 1:42-52.

103. Mackenbach JD, Lakerveld J, van Lenthe FJ, Kawachi I, McKee M, Rutter H, et al. Neighbourhood social capital: measurement issues and associations with health outcomes. Obesity reviews : an official journal of the International Association for the Study of Obesity. 2016;17 Suppl 1:96-107.

104. Macniven R, Richards J, Gubhaju L, Joshy G, Bauman A, Banks E, et al. Physical activity, healthy lifestyle behaviors, neighborhood environment characteristics and social support among Australian Aboriginal and non-Aboriginal adults. Prev Med Rep. 2016;3:203-10.

105. Maisel JL. Impact of Older Adults' Neighborhood Perceptions on Walking Behavior. Journal of aging and physical activity. 2016;24(2):247-55.

106. Marlier M, Van Dyck D, Cardon G, De Bourdeaudhuij I, Babiak K, Willem A. Interrelation of Sport Participation, Physical Activity, Social Capital and Mental Health in Disadvantaged Communities: A SEM-Analysis. PLoS One. 2015;10(10):e0140196.

107. Mason P, Kearns A, Livingston M. "Safe Going": the influence of crime rates and perceived crime and safety on walking in deprived neighbourhoods. Soc Sci Med. 2013;91:15-24.

108. McDonald NC. Travel and the social environment: Evidence from Alameda County, California. Transportation Research Part D: Transport and Environment. 2007;12(1):53-63.

109. McDonald NC. The effect of objectively measured crime on walking in minority adults. Am J Health Promot. 2008;22(6):433-6.

110. McGinn AP, Evenson KR, Herring AH, Huston SL, Rodriguez DA. The association of perceived and objectively measured crime with physical activity: a cross-sectional analysis. J Phys Act Health. 2008;5(1):117-31.

111. Mendes de Leon CF, Cagney KA, Bienias JL, Barnes LL, Skarupski KA, Scherr PA, et al. Neighborhood social cohesion and disorder in relation to walking in community-dwelling older adults: a multilevel analysis. J Aging Health. 2009;21(1):155-71.

112. Merom D, Bauman A, Phongsavan P, Cerin E, Kassis M, Brown W, et al. Can a motivational intervention overcome an unsupportive environment for walking--findings from the Step-by-Step Study. Annals of behavioral medicine : a publication of the Society of Behavioral Medicine. 2009;38(2):137-46.

113. Mohnen SM, Volker B, Flap H, Groenewegen PP. Health-related behavior as a mechanism behind the relationship between neighborhood social capital and individual health--a multilevel analysis. BMC Public Health. 2012;12:116.

114. Mota J, Lacerda A, Santos MP, Ribeiro JC, Carvalho J. Perceived neighborhood environments and physical activity in an elderly sample. Perceptual and motor skills. 2007;104(2):438-44.

115. Mota J, Ribeiro JC, Santos MP. Obese girls differences in neighbourhood perceptions, screen time and socioeconomic status according to level of physical activity. Health education research. 2009;24(1):98-104.

116. Mota J, Almeida M, Santos R, Ribeiro JC, Santos MP. Association of perceived environmental characteristics and participation in organized and non-organized physical activities of adolescents. Pediatric exercise science. 2009;21(2):233-9.

117. Motl RW, Dishman RK, Saunders RP, Dowda M, Pate RR. Perceptions of physical and social environment variables and self-efficacy as correlates of self-reported physical activity among adolescent girls. J Pediatr Psychol. 2007;32(1):6-12.

118. Muthuri SK, Wachira LJ, Onywera VO, Tremblay MS. Associations Between Parental Perceptions of the Neighborhood Environment and Childhood Physical Activity: Results from ISCOLE-Kenya. J Phys Act Health. 2016;13(3):333-43.

119. Nehme EK, Oluyomi AO, Calise TV, Kohl HW, 3rd. Environmental Correlates of Recreational Walking in the Neighborhood. Am J Health Promot. 2016;30(3):139-48.

120. Noonan RJ, Boddy LM, Knowles ZR, Fairclough SJ. Cross-sectional associations between high-deprivation home and neighbourhood environments, and health-related variables among Liverpool children. BMJ Open. 2016;6(1):e008693.

121. Oh AY, Zenk SN, Wilbur J, Block R, McDevitt J, Wang E. Effects of perceived and objective neighborhood crime on walking frequency among midlife African American women in a home-based walking intervention. J Phys Act Health. 2010;7(4):432-41.

122. Oluyomi AO, Lee C, Nehme E, Dowdy D, Ory MG, Hoelscher DM. Parental safety concerns and active school commute: correlates across multiple domains in the home-to-school journey. The international journal of behavioral nutrition and physical activity. 2014;11(1):32.

123. Osypuk TL, Diez Roux AV, Hadley C, Kandula NR. Are immigrant enclaves healthy places to live? The Multi-ethnic Study of Atherosclerosis. Soc Sci Med. 2009;69(1):110-20.

124. Oyeyemi AL, Adegoke BO, Sallis JF, Oyeyemi AY, De Bourdeaudhuij I. Perceived crime and traffic safety is related to physical activity among adults in Nigeria. BMC Public Health. 2012;12:294.

125. Oyeyemi AY, Akinrolie O, Oyeyemi AL. Health-related physical activity is associated with perception of environmental hygiene and safety among adults in low-income neighbourhoods in Nigeria. European Journal of Physiotherapy. 2015;17(1):45-53.

126. Pabayo R, Molnar BE, Cradock A, Kawachi I. The relationship between neighborhood socioeconomic characteristics and physical inactivity among adolescents living in Boston, Massachusetts. Am J Public Health. 2014;104(11):e142-9.

127. Peachey AA, Baller SL. Perceived Built Environment Characteristics of On-Campus and Off-Campus Neighborhoods Associated With Physical Activity of College Students. Journal of American college health : J of ACH. 2015:0.

128. Pelclova J, Fromel K, Cuberek R. Gender-specific associations between perceived neighbourhood walkability and meeting walking recommendations when walking for transport and recreation for Czech inhabitants over 50 years of age. Int J Environ Res Public Health. 2014;11(1):527-36.

129. Perez LG, Slymen DJ, Sallis JF, Ayala GX, Elder JP, Arredondo EM. Interactions between individual and perceived environmental factors on Latinas' physical activity. J Public Health (Oxf). 2017;39(2):e10-e8.

130. Piro FN, Noss O, Claussen B. Physical activity among elderly people in a city population: the influence of neighbourhood level violence and self perceived safety. Journal of epidemiology and community health. 2006;60(7):626-32.

131. Quon EC, McGrath JJ. Community, Family, and Subjective Socioeconomic Status: Relative Status and Adolescent Health. Health psychology : official journal of the Division of Health Psychology, American Psychological Association. 2014.

132. Rech CR, Reis RS, Hino AA, Hallal PC. Personal, social and environmental correlates of physical activity in adults from Curitiba, Brazil. Prev Med. 2014;58:53-7.

133. Reis RS, Yan Y, Parra DC, Brownson RC. Assessing participation in community-based physical activity programs in Brazil. Medicine and science in sports and exercise. 2014;46(1):92-8.

134. Remmers T, Van Kann D, Gubbels J, Schmidt S, de Vries S, Ettema D, et al. Moderators of the longitudinal relationship between the perceived physical environment and outside play in children: the KOALA birth cohort study. The international journal of behavioral nutrition and physical activity. 2014;11(1):150.

135. Rhodes RE, Brown SG, McIntyre CA. Integrating the perceived neighborhood environment and the theory of planned behavior when predicting walking in a Canadian adult sample. Am J Health Promot. 2006;21(2):110-8.

136. Ribeiro AI, Pires A, Carvalho MS, Pina MF. Distance to parks and non-residential destinations influences physical activity of older people, but crime doesn't: a cross-sectional study in a southern European city. BMC Public Health. 2015;15:593.

137. Rind E, Shortt N, Mitchell R, Richardson EA, Pearce J. Are income-related differences in active travel associated with physical environmental characteristics? A multi-level ecological approach. The international journal of behavioral nutrition and physical activity. 2015;12:73.

138. Robinson AI, Carnes F, Oreskovic NM. Spatial analysis of crime incidence and adolescent physical activity. Prev Med. 2016;85:74-7.

139. Rossen LM, Pollack KM, Curriero FC, Shields TM, Smart MJ, Furr-Holden CD, et al. Neighborhood incivilities, perceived neighborhood safety, and walking to school among urban-dwelling children. J Phys Act Health. 2011;8(2):262-71.

140. Salahuddin M, Nehme E, Ranjit N, Kim YJ, Oluyomi AO, Dowdy D, et al. Does Parents' Social Cohesion Influence Their Perception of Neighborhood Safety and Their Children's Active Commuting to and From School? J Phys Act Health. 2016;13(12):1301-9.

141. Sallis JF, King AC, Sirard JR, Albright CL. Perceived environmental predictors of physical activity over 6 months in adults: activity counseling trial. Health psychology : official journal of the Division of Health Psychology, American Psychological Association. 2007;26(6):701-9.

142. Salmon J, Veitch J, Abbott G, Chin AM, Brug JJ, teVelde SJ, et al. Are associations between the perceived home and neighbourhood environment and children's physical activity and sedentary behaviour moderated by urban/rural location? Health & place. 2013;24:44-53.

143. Samuel LJ, Dennison Himmelfarb CR, Szklo M, Seeman TE, Echeverria SE, Diez Roux AV. Social engagement and chronic disease risk behaviors: The Multi-Ethnic Study of Atherosclerosis. Prev Med. 2015;71:61-6.

144. Santana P, Santos R, Nogueira H. The link between local environment and obesity: a multilevel analysis in the Lisbon Metropolitan Area, Portugal. Soc Sci Med. 2009;68(4):601-9.

145. Santos R, Silva P, Santos P, Ribeiro JC, Mota J. Physical activity and perceived environmental attributes in a sample of Portuguese adults: results from the Azorean Physical Activity and Health study. Prev Med. 2008;47(1):83-8.

146. Santos MS, Vale MS, Miranda L, Mota J. Socio-demographic and perceived environmental correlates of walking in Portuguese adults--a multilevel analysis. Health & place. 2009;15(4):1094-9.

147. Schoeny ME, Fogg L, Buchholz SW, Miller A, Wilbur J. Barriers to physical activity as moderators of intervention effects. Prev Med Rep. 2017;5:57-64.

148. Schulz A, Mentz G, Johnson-Lawrence V, Israel BA, Max P, Zenk SN, et al. Independent and joint associations between multiple measures of the built and social environment and physical activity in a multi-ethnic urban community. J Urban Health. 2013;90(5):872-87.

149. Shenassa ED, Liebhaber A, Ezeamama A. Perceived safety of area of residence and exercise: a pan-European study. Am J Epidemiol. 2006;163(11):1012-7.

150. Siceloff ER, Coulon SM, Wilson DK. Physical activity as a mediator linking neighborhood environmental supports and obesity in African Americans in the path trial. Health psychology : official journal of the Division of Health Psychology, American Psychological Association. 2014;33(5):481-9.

151. Singh GK, Kogan MD, Siahpush M, van Dyck PC. Independent and joint effects of socioeconomic, behavioral, and neighborhood characteristics on physical inactivity and activity levels among US children and adolescents. J Community Health. 2008;33(4):206-16.

152. Singh GK, Kogan MD, Siahpush M, van Dyck PC. Prevalence and correlates of state and regional disparities in vigorous physical activity levels among US children and adolescents. J Phys Act Health. 2009;6(1):73-87.

153. Solomon E, Rees T, Ukoumunne OC, Metcalf B, Hillsdon M. Personal, social, and environmental correlates of physical activity in adults living in rural south-west England: a cross-sectional analysis. The international journal of behavioral nutrition and physical activity. 2013;10:129.

154. Soltero EG, Hernandez DC, O'Connor DP, Lee RE. Does social support mediate the relationship among neighborhood disadvantage, incivilities, crime and physical activity? Prev Med. 2015;72:44-9.

155. Stimpson JP, Nash AC, Ju H, Eschbach K. Neighborhood Deprivation is associated with lower levels of serum carotenoids among adults participating in the Third National Health and Nutrition Examination Survey. Journal of the American Dietetic Association. 2007;107(11):1895-902.

156. Strath SJ, Greenwald MJ, Isaacs R, Hart TL, Lenz EK, Dondzila CJ, et al. Measured and perceived environmental characteristics are related to accelerometer defined physical activity in older adults. The international journal of behavioral nutrition and physical activity. 2012;9:40.

157. Strong LL, Reitzel LR, Wetter DW, McNeill LH. Associations of perceived neighborhood physical and social environments with physical activity and television viewing in African-American men and women. Am J Health Promot. 2013;27(6):401-9.

158. Sugiyama T, Howard NJ, Paquet C, Coffee NT, Taylor AW, Daniel M. Do Relationships Between Environmental Attributes and Recreational Walking Vary According to Area-Level Socioeconomic Status? J Urban Health. 2015.

159. Sugiyama T, Shibata A, Koohsari MJ, Tanamas SK, Oka K, Salmon J, et al. Neighborhood environmental attributes and adults' maintenance of regular walking. Medicine and science in sports and exercise. 2015;47(6):1204-10.

160. Tanaka C, Naruse T, Taguchi A, Nagata S, Arimoto A, Ohashi Y, et al. Conformity to the neighborhood modifies the association between recreational walking and social norms among middle-aged Japanese people. Jpn J Nurs Sci. 2016;13(4):451-65.

161. Tappe KA, Glanz K, Sallis JF, Zhou C, Saelens BE. Children's physical activity and parents' perception of the neighborhood environment: neighborhood impact on kids study. The international journal of behavioral nutrition and physical activity. 2013;10:39.

162. Timperio A, Ball K, Salmon J, Roberts R, Giles-Corti B, Simmons D, et al. Personal, family, social, and environmental correlates of active commuting to school. Am J Prev Med. 2006;30(1):45-51.

163. Timperio A, Veitch J, Carver A. Safety in numbers: Does perceived safety mediate associations between the neighborhood social environment and physical activity among women living in disadvantaged neighborhoods? Prev Med. 2015.

164. Troped PJ, Tamura K, Whitcomb HA, Laden F. Perceived built environment and physical activity in U.S. women by sprawl and region. Am J Prev Med. 2011;41(5):473-9.

165. Turrell G, Haynes M, Burton NW, Giles-Corti B, Oldenburg B, Wilson LA, et al. Neighborhood disadvantage and physical activity: baseline results from the HABITAT multilevel longitudinal study. Ann Epidemiol. 2010;20(3):171-81.

166. Ueshima K, Fujiwara T, Takao S, Suzuki E, Iwase T, Doi H, et al. Does social capital promote physical activity? A population-based study in Japan. PLoS One. 2010;5(8):e12135.

167. Vamos CA, Sun H, Flory SB, DeBate R, Daley EM, Thompson E, et al. Community Level Predictors of Physical Activity Among Women in the Preconception Period. Maternal and child health journal. 2015.

168. Van Cauwenberg J, De Donder L, Clarys P, De Bourdeaudhuij I, Buffel T, De Witte N, et al. Relationships between the perceived neighborhood social environment and walking for transportation among older adults. Soc Sci Med. 2014;104:23-30.

169. Van Dyck D, Cerin E, De Bourdeaudhuij I, Salvo D, Christiansen LB, Macfarlane D, et al. Moderating effects of age, gender and education on the associations of perceived neighborhood environment attributes with accelerometer-based physical activity: The IPEN adult study. Health & place. 2015;36:65-73.

170. Van Holle V, Van Cauwenberg J, Gheysen F, Van Dyck D, Deforche B, Van de Weghe N, et al. The Association between Belgian Older Adults' Physical Functioning and Physical Activity: What Is the Moderating Role of the Physical Environment? PLoS One. 2016;11(2):e0148398.

171. van Loon J, Frank LD, Nettlefold L, Naylor PJ. Youth physical activity and the neighbourhood environment: examining correlates and the role of neighbourhood definition. Soc Sci Med. 2014;104:107-15.

172. Vanwolleghem G, Van Dyck D, De Meester F, De Bourdeaudhuij I, Cardon G, Gheysen F. Which Socio-Ecological Factors Associate with a Switch to or Maintenance of Active and Passive Transport during the Transition from Primary to Secondary School? PLoS One. 2016;11(5):e0156531.

173. Verhoeven H, Simons D, Van Dyck D, Van Cauwenberg J, Clarys P, De Bourdeaudhuij I, et al. Psychosocial and Environmental Correlates of Walking, Cycling, Public Transport and Passive Transport to Various Destinations in Flemish Older Adolescents. PLoS One. 2016;11(1):e0147128.

174. Voorhees CC, Catellier DJ, Ashwood JS, Cohen DA, Rung A, Lytle L, et al. Neighborhood socioeconomic status and non school physical activity and body mass index in adolescent girls. J Phys Act Health. 2009;6(6):731-40.

175. Wang Z, Lee C. Site and neighborhood environments for walking among older adults. Health & place. 2010;16(6):1268-79.

176. Weber Corseuil M, Hallal PC, Xavier Corseuil H, Jayce Ceola Schneider I, d'Orsi E. Safety from crime and physical activity among older adults: a population-based study in Brazil. Journal of environmental and public health. 2012;2012:641010.

177. Weir LA, Etelson D, Brand DA. Parents' perceptions of neighborhood safety and children's physical activity. Prev Med. 2006;43(3):212-7.

178. Wen M, Kandula NR, Lauderdale DS. Walking for transportation or leisure: what difference does the neighborhood make? Journal of general internal medicine. 2007;22(12):1674-80.

179. Wen M, Zhang X. Contextual effects of built and social environments of urban neighborhoods on exercise: a multilevel study in Chicago. Am J Health Promot. 2009;23(4):247-54.

180. Zandieh R, Martinez J, Flacke J, Jones P, van Maarseveen M. Older Adults' Outdoor Walking: Inequalities in Neighbourhood Safety, Pedestrian Infrastructure and Aesthetics. Int J Environ Res Public Health. 2016;13(12).

181. Zhu X, Arch B, Lee C. Personal, social, and environmental correlates of walking to school behaviors: case study in Austin, Texas. TheScientificWorldJournal. 2008;8:859-72.
