# Supplementary material for: The neighborhood social environment and physical activity: a systematic scoping review
Source: Int J Behav Nutr Phys Act. 2019 Dec 9;16:124. doi: 10.1186/s12966-019-0873-7 (PMC6902518; doi:10.1186/s12966-019-0873-7)
Supplement: Supplementary file 4 — Additional file 4: Table S2. Characteristics of articles reviewed (n=181). [file 12966_2019_873_MOESM4_ESM.docx]

| **Table S2. Characteristics of articles reviewed (n=181)** | | |
| --- | --- | --- |
| **Characteristics** | **# of Articles** | **Reference #** |
| **Total Sample Size** |  |  |
| <100 | 4 | [1-4] |
| 100-249 | 16 | [5-20] |
| 250-499 | 35 | [21-55] |
| 500-1999 | 60 | [56-115] |
| 2000-4999 | 25 | [116-140] |
| ≥5000 | 40 | [141-180] |
| Not reported | 1 | [181] |
| **Study Design** |  |  |
| Cross-sectional | 166 | [1, 3-21, 23-26, 28-32, 34-46, 48-66, 68, 69, 71-90, 92-94, 96, 97, 99-118, 120-133, 135, 137-145, 147-175, 177-181] |
| Longitudinal | 10 | [22, 47, 70, 91, 98, 119, 134, 136, 146, 176] |
| Experimental | 4 | [2, 27, 33, 67] |
| Multiple designs | 1 | [95] |
| **Age Group^1^** |  |  |
| Youth | 62 | [2-5, 7, 8, 10, 11, 14, 16, 21-23, 28, 32, 35, 37, 38, 41, 46, 49, 53, 56, 59-61, 64, 68, 70, 76-78, 80, 81, 85, 87-90, 92, 96, 99, 101, 102, 104, 105, 107, 109-111, 121, 128, 131, 141, 142, 144, 146, 150, 158, 160, 172, 173] |
| Adult | 106 | [1, 6, 9, 12, 13, 15, 17-20, 24-27, 29-31, 33, 34, 36, 39, 40, 42-45, 47, 48, 50-52, 54, 55, 57, 58, 62, 63, 65, 67, 69, 71, 74, 75, 79, 82-84, 86, 91, 93-95, 97, 98, 100, 103, 108, 112-120, 122-124, 127, 129, 130, 132, 133, 135-140, 143, 147-149, 151, 153, 154, 157, 159, 161-165, 167-171, 175-181] |
| Both | 12 | [66, 72, 73, 106, 125, 126, 134, 145, 152, 155, 166, 174] |
| Not reported | 1 | [156] |
| **Study Setting** |  |  |
| Urban | 105 | [1-7, 10, 11, 13-15, 17, 20, 21, 24, 25, 27, 28, 30, 31, 35-39, 41-44, 46-48, 51, 52, 54, 56-61, 64-66, 69, 71-75, 77, 80, 85, 87-89, 91, 92, 95-102, 104, 105, 108, 110, 112-118, 124-126, 129-132, 138-142, 147-149, 151, 153, 156, 157, 161, 162, 165-168, 179, 181] |
| Rural | 4 | [40, 62, 106, 120] |
| Both | 21 | [12, 45, 49, 68, 90, 94, 103, 107, 109, 111, 133, 135, 146, 152, 155, 169-173, 178] |
| Not Reported/Unclear | 51 | [8, 9, 16, 18, 19, 22, 23, 26, 29, 32-34, 50, 53, 55, 63, 67, 70, 76, 78, 79, 81-84, 86, 93, 119, 121-123, 127, 128, 134, 136, 137, 143-145, 150, 154, 158-160, 163, 164, 174-177, 180] |
| **Geographic Origin** |  |  |
| Africa | 3 | [17, 74, 77] |
| Asia | 8 | [54, 62, 83, 85, 94, 117, 129, 132] |
| Australia/New Zealand | 19 | [1, 33, 41, 47, 49, 60, 63, 86, 98, 109, 113, 118, 119, 130, 140, 143, 152, 163, 176] |
| Europe | 46 | [6, 14, 16, 20, 22, 24, 32, 36, 53, 55, 59, 69, 70, 78, 81, 103, 104, 111, 112, 120, 122, 123, 125, 127, 137, 139, 141, 142, 145, 149, 154-162, 164, 166, 168-171, 178] |
| North America | 94 | [2-5, 7-12, 15, 18, 19, 21, 23, 25-31, 34, 35, 37-40, 42-46, 48, 50-52, 56, 58, 61, 64-68, 72, 73, 75, 76, 78-80, 84, 88-93, 95-97, 99-102, 105-108, 110, 115, 116, 121, 124, 126, 128, 131, 133-135, 144, 146, 148, 150, 151, 167, 172-175, 177, 180, 181] |
| South America | 9 | [57, 71, 82, 87, 114, 136, 138, 153, 165] |
| Multiple origins | 2 | [147, 179] |
| **Year of Publication** |  |  |
| 2005-2009 | 52 | [1, 7-9, 11, 13, 14, 21, 29, 32-34, 37, 41, 47, 53, 56, 58, 60, 67, 75, 78-80, 85, 92, 95, 97, 99, 101, 111, 116, 122, 124, 126-128, 140, 144, 148, 149, 151, 154, 156, 168-174, 180] |
| 2010-2014 | 70 | [5, 6, 17-19, 23, 26, 28, 31, 35, 36, 39, 42-46, 49-51, 57, 61, 64-66, 70, 71, 73, 76, 81, 83, 86, 89, 93, 94, 100, 105, 106, 108, 110, 113, 114, 117, 120, 121, 123, 125, 130, 132, 134-137, 139, 141, 142, 145-147, 150, 152, 157, 158, 160, 164, 165, 175, 176, 178, 181] |
| 2015-2018 | 59 | [2-4, 10, 12, 15, 16, 20, 22, 24, 25, 27, 30, 38, 40, 48, 52, 54, 55, 59, 62, 63, 68, 69, 72, 74, 77, 82, 84, 87, 88, 90, 91, 96, 98, 102-104, 107, 109, 112, 115, 118, 119, 129, 131, 133, 138, 143, 153, 155, 159, 161-163, 166, 167, 177, 179] |
| **Physical Activity Measures^2^** |  |  |
| Objective | 31 | [2-4, 6-8, 10, 17, 18, 20, 23, 24, 26, 27, 30, 31, 33, 35, 38, 40, 41, 46, 49, 58, 61, 66, 77, 88, 99, 104, 179] |
| Subjective | 150 | [1, 5, 9, 11-16, 19, 21, 22, 25, 28, 29, 32, 34, 36, 37, 39, 42-45, 47, 48, 50-57, 59, 60, 62-65, 67-76, 78-87, 89-98, 100-103, 105-178, 180, 181] |
| ^1^ Sample age was categorized as youth (≤18 year of age), adult (>18 year of age), or both.  ^2^Physical activity was considered to be objectively-measured if a device (i.e. accelerometer, pedometer, heart rate monitor, etc.) was used to measure physical activity. Subjectively-measured physical activity included self- or parent-reports using a survey instrument. | | |

1. Annear, M.J., G. Cushman, and B. Gidlow, *Leisure time physical activity differences among older adults from diverse socioeconomic neighborhoods.* Health Place, 2009. **15**(2): p. 482-90.

2. Broyles, S.T., et al., *The Influence of Neighborhood Crime on Increases in Physical Activity during a Pilot Physical Activity Intervention in Children.* J Urban Health, 2016. **93**(2): p. 271-8.

3. Cerin, E., et al., *Places where preschoolers are (in)active: an observational study on Latino preschoolers and their parents using objective measures.* Int J Behav Nutr Phys Act, 2016. **13**: p. 29.

4. Robinson, A.I., F. Carnes, and N.M. Oreskovic, *Spatial analysis of crime incidence and adolescent physical activity.* Prev Med, 2016. **85**: p. 74-7.

5. Echeverria, S.E., et al., *A community survey on neighborhood violence, park use, and physical activity among urban youth.* J Phys Act Health, 2014. **11**(1): p. 186-94.

6. Fox, K.R., et al., *Neighbourhood deprivation and physical activity in UK older adults.* Health Place, 2011. **17**(2): p. 633-40.

7. Jago, R., T. Baranowski, and J.C. Baranowski, *Observed, GIS, and self-reported environmental features and adolescent physical activity.* Am J Health Promot, 2006. **20**(6): p. 422-8.

8. Jago, R., T. Baranowski, and M. Harris, *Relationships Between GIS Environmental Features and Adolescent Male Physical Activity: GIS Coding Differences.* J Phys Act Health, 2006. **3**(2): p. 230-242.

9. King, D., *Neighborhood and individual factors in activity in older adults: results from the neighborhood and senior health study.* J Aging Phys Act, 2008. **16**(2): p. 144-70.

10. Kneeshaw-Price, S.H., et al., *Neighborhood Crime-Related Safety and Its Relation to Children's Physical Activity.* J Urban Health, 2015. **92**(3): p. 472-89.

11. Kuo, J., et al., *Associations between family support, family intimacy, and neighborhood violence and physical activity in urban adolescent girls.* Am J Public Health, 2007. **97**(1): p. 101-3.

12. Maisel, J.L., *Impact of Older Adults' Neighborhood Perceptions on Walking Behavior.* J Aging Phys Act, 2016. **24**(2): p. 247-55.

13. Mota, J., et al., *Perceived neighborhood environments and physical activity in an elderly sample.* Percept Mot Skills, 2007. **104**(2): p. 438-44.

14. Mota, J., J.C. Ribeiro, and M.P. Santos, *Obese girls differences in neighbourhood perceptions, screen time and socioeconomic status according to level of physical activity.* Health Educ Res, 2009. **24**(1): p. 98-104.

15. Nehme, E.K., et al., *Environmental Correlates of Recreational Walking in the Neighborhood.* Am J Health Promot, 2016. **30**(3): p. 139-48.

16. Noonan, R.J., et al., *Cross-sectional associations between high-deprivation home and neighbourhood environments, and health-related variables among Liverpool children.* BMJ Open, 2016. **6**(1): p. e008693.

17. Oyeyemi, A.L., et al., *Perceived crime and traffic safety is related to physical activity among adults in Nigeria.* BMC Public Health, 2012. **12**: p. 294.

18. Strath, S.J., et al., *Measured and perceived environmental characteristics are related to accelerometer defined physical activity in older adults.* Int J Behav Nutr Phys Act, 2012. **9**: p. 40.

19. Wang, Z. and C. Lee, *Site and neighborhood environments for walking among older adults.* Health Place, 2010. **16**(6): p. 1268-79.

20. Zandieh, R., et al., *Older Adults' Outdoor Walking: Inequalities in Neighbourhood Safety, Pedestrian Infrastructure and Aesthetics.* Int J Environ Res Public Health, 2016. **13**(12).

21. Weir, L.A., D. Etelson, and D.A. Brand, *Parents' perceptions of neighborhood safety and children's physical activity.* Prev Med, 2006. **43**(3): p. 212-7.

22. Vanwolleghem, G., et al., *Which Socio-Ecological Factors Associate with a Switch to or Maintenance of Active and Passive Transport during the Transition from Primary to Secondary School?* PLoS One, 2016. **11**(5): p. e0156531.

23. van Loon, J., et al., *Youth physical activity and the neighbourhood environment: examining correlates and the role of neighbourhood definition.* Soc Sci Med, 2014. **104**: p. 107-15.

24. Van Holle, V., et al., *The Association between Belgian Older Adults' Physical Functioning and Physical Activity: What Is the Moderating Role of the Physical Environment?* PLoS One, 2016. **11**(2): p. e0148398.

25. Soltero, E.G., et al., *Does social support mediate the relationship among neighborhood disadvantage, incivilities, crime and physical activity?* Prev Med, 2015. **72**: p. 44-9.

26. Siceloff, E.R., S.M. Coulon, and D.K. Wilson, *Physical activity as a mediator linking neighborhood environmental supports and obesity in African Americans in the path trial.* Health Psychol, 2014. **33**(5): p. 481-9.

27. Schoeny, M.E., et al., *Barriers to physical activity as moderators of intervention effects.* Prev Med Rep, 2017. **5**: p. 57-64.

28. Rossen, L.M., et al., *Neighborhood incivilities, perceived neighborhood safety, and walking to school among urban-dwelling children.* J Phys Act Health, 2011. **8**(2): p. 262-71.

29. Rhodes, R.E., S.G. Brown, and C.A. McIntyre, *Integrating the perceived neighborhood environment and the theory of planned behavior when predicting walking in a Canadian adult sample.* Am J Health Promot, 2006. **21**(2): p. 110-8.

30. Perez, L.G., et al., *Interactions between individual and perceived environmental factors on Latinas' physical activity.* J Public Health (Oxf), 2017. **39**(2): p. e10-e18.

31. Oh, A.Y., et al., *Effects of perceived and objective neighborhood crime on walking frequency among midlife African American women in a home-based walking intervention.* J Phys Act Health, 2010. **7**(4): p. 432-41.

32. Mota, J., et al., *Association of perceived environmental characteristics and participation in organized and non-organized physical activities of adolescents.* Pediatr Exerc Sci, 2009. **21**(2): p. 233-9.

33. Merom, D., et al., *Can a motivational intervention overcome an unsupportive environment for walking--findings from the Step-by-Step Study.* Ann Behav Med, 2009. **38**(2): p. 137-46.

34. McDonald, N.C., *The effect of objectively measured crime on walking in minority adults.* Am J Health Promot, 2008. **22**(6): p. 433-6.

35. Lovasi, G.S., et al., *Is the environment near home and school associated with physical activity and adiposity of urban preschool children?* J Urban Health, 2011. **88**(6): p. 1143-57.

36. Kremers, S.P., et al., *Associations between safety from crime, cycling, and obesity in a Dutch elderly population: results from the Longitudinal Aging Study Amsterdam.* J Environ Public Health, 2012. **2012**: p. 127857.

37. Kerr, J., et al., *Active commuting to school: Associations with environment and parental concerns.* Med Sci Sports Exerc, 2006. **38**(4): p. 787-94.

38. Katapally, T.R. and N. Muhajarine, *Capturing the Interrelationship between Objectively Measured Physical Activity and Sedentary Behaviour in Children in the Context of Diverse Environmental Exposures.* Int J Environ Res Public Health, 2015. **12**(9): p. 10995-1011.

39. Kaczynski, A.T. and T.D. Glover, *Talking the talk, walking the walk: examining the effect of neighbourhood walkability and social connectedness on physical activity.* J Public Health (Oxf), 2012. **34**(3): p. 382-9.

40. Jilcott Pitts, S.B., et al., *Associations between neighborhood-level factors related to a healthful lifestyle and dietary intake, physical activity, and support for obesity prevention polices among rural adults.* J Community Health, 2015. **40**(2): p. 276-84.

41. Hume, C., J. Salmon, and K. Ball, *Associations of children's perceived neighborhood environments with walking and physical activity.* Am J Health Promot, 2007. **21**(3): p. 201-7.

42. Halbert, C.H., et al., *Collective efficacy and obesity-related health behaviors in a community sample of African Americans.* J Community Health, 2014. **39**(1): p. 124-31.

43. Gay, J.L., R.P. Saunders, and M. Dowda, *The relationship of physical activity and the built environment within the context of self-determination theory.* Ann Behav Med, 2011. **42**(2): p. 188-96.

44. Gallagher, N.A., P.J. Clarke, and K.A. Gretebeck, *Gender differences in neighborhood walking in older adults.* J Aging Health, 2014. **26**(8): p. 1280-300.

45. Edwards, M. and G. Cunningham, *Examining the associations of perceived community racism with self-reported physical activity levels and health among older racial minority adults.* J Phys Act Health, 2013. **10**(7): p. 932-9.

46. Durand, C.P., et al., *Does community type moderate the relationship between parent perceptions of the neighborhood and physical activity in children?* Am J Health Promot, 2012. **26**(6): p. 371-80.

47. Cleland, V.J., A. Timperio, and D. Crawford, *Are perceptions of the physical and social environment associated with mothers' walking for leisure and for transport? A longitudinal study.* Prev Med, 2008. **47**(2): p. 188-93.

48. Chaudhury, H., et al., *Neighbourhood environment and physical activity in older adults.* Soc Sci Med, 2016. **149**: p. 104-13.

49. Carver, A., et al., *Are children and adolescents less active if parents restrict their physical activity and active transport due to perceived risk?* Soc Sci Med, 2010. **70**(11): p. 1799-805.

50. Bungum, T.J., et al., *Perceived environmental physical activity correlates among Asian Pacific Islander Americans.* J Phys Act Health, 2012. **9**(8): p. 1098-104.

51. Armstrong-Brown, J., et al., *Redefining Racial Residential Segregation and its Association With Physical Activity Among African Americans 50 Years and Older: A Mixed Methods Approach.* J Aging Phys Act, 2014.

52. Andersen, L., J. Gustat, and A.B. Becker, *The Relationship Between the Social Environment and Lifestyle-Related Physical Activity in a Low-Income African American Inner-City Southern Neighborhood.* Journal of Community Health, 2015. **40**(5): p. 967-974.

53. Alton, D., et al., *Relationship between walking levels and perceptions of the local neighbourhood environment.* Arch Dis Child, 2007. **92**(1): p. 29-33.

54. Adlakha, D., et al., *"Can we walk?" Environmental supports for physical activity in India.* Prev Med, 2017. **103s**: p. S81-s89.

55. Marlier, M., et al., *Interrelation of Sport Participation, Physical Activity, Social Capital and Mental Health in Disadvantaged Communities: A SEM-Analysis.* PLoS One, 2015. **10**(10): p. e0140196.

56. Zhu, X., B. Arch, and C. Lee, *Personal, social, and environmental correlates of walking to school behaviors: case study in Austin, Texas.* ScientificWorldJournal, 2008. **8**: p. 859-72.

57. Weber Corseuil, M., et al., *Safety from crime and physical activity among older adults: a population-based study in Brazil.* J Environ Public Health, 2012. **2012**: p. 641010.

58. Voorhees, C.C., et al., *Neighborhood socioeconomic status and non school physical activity and body mass index in adolescent girls.* J Phys Act Health, 2009. **6**(6): p. 731-40.

59. Verhoeven, H., et al., *Psychosocial and Environmental Correlates of Walking, Cycling, Public Transport and Passive Transport to Various Destinations in Flemish Older Adolescents.* PLoS One, 2016. **11**(1): p. e0147128.

60. Timperio, A., et al., *Personal, family, social, and environmental correlates of active commuting to school.* Am J Prev Med, 2006. **30**(1): p. 45-51.

61. Tappe, K.A., et al., *Children's physical activity and parents' perception of the neighborhood environment: neighborhood impact on kids study.* Int J Behav Nutr Phys Act, 2013. **10**: p. 39.

62. Tanaka, C., et al., *Conformity to the neighborhood modifies the association between recreational walking and social norms among middle-aged Japanese people.* Japan Journal of Nursing Science, 2016. **13**(4): p. 451-465.

63. Sugiyama, T., et al., *Do Relationships Between Environmental Attributes and Recreational Walking Vary According to Area-Level Socioeconomic Status?* J Urban Health, 2015.

64. Strong, L.L., et al., *Associations of perceived neighborhood physical and social environments with physical activity and television viewing in African-American men and women.* Am J Health Promot, 2013. **27**(6): p. 401-9.

65. Schulz, A., et al., *Independent and joint associations between multiple measures of the built and social environment and physical activity in a multi-ethnic urban community.* J Urban Health, 2013. **90**(5): p. 872-87.

66. Salmon, J., et al., *Are associations between the perceived home and neighbourhood environment and children's physical activity and sedentary behaviour moderated by urban/rural location?* Health Place, 2013. **24**: p. 44-53.

67. Sallis, J.F., et al., *Perceived environmental predictors of physical activity over 6 months in adults: activity counseling trial.* Health Psychol, 2007. **26**(6): p. 701-9.

68. Salahuddin, M., et al., *Does Parents' Social Cohesion Influence Their Perception of Neighborhood Safety and Their Children's Active Commuting to and From School?* J Phys Act Health, 2016. **13**(12): p. 1301-1309.

69. Ribeiro, A.I., et al., *Distance to parks and non-residential destinations influences physical activity of older people, but crime doesn't: a cross-sectional study in a southern European city.* BMC Public Health, 2015. **15**: p. 593.

70. Remmers, T., et al., *Moderators of the longitudinal relationship between the perceived physical environment and outside play in children: the KOALA birth cohort study.* Int J Behav Nutr Phys Act, 2014. **11**(1): p. 150.

71. Rech, C.R., et al., *Personal, social and environmental correlates of physical activity in adults from Curitiba, Brazil.* Prev Med, 2014. **58**: p. 53-7.

72. Peachey, A.A. and S.L. Baller, *Perceived Built Environment Characteristics of On-Campus and Off-Campus Neighborhoods Associated With Physical Activity of College Students.* J Am Coll Health, 2015: p. 0.

73. Pabayo, R., et al., *The relationship between neighborhood socioeconomic characteristics and physical inactivity among adolescents living in Boston, Massachusetts.* Am J Public Health, 2014. **104**(11): p. e142-9.

74. Oyeyemi, A.Y., O. Akinrolie, and A.L. Oyeyemi, *Health-related physical activity is associated with perception of environmental hygiene and safety among adults in low-income neighbourhoods in Nigeria.* European Journal of Physiotherapy, 2015. **17**(1): p. 45-53.

75. Osypuk, T.L., et al., *Are immigrant enclaves healthy places to live? The Multi-ethnic Study of Atherosclerosis.* Soc Sci Med, 2009. **69**(1): p. 110-20.

76. Oluyomi, A.O., et al., *Parental safety concerns and active school commute: correlates across multiple domains in the home-to-school journey.* Int J Behav Nutr Phys Act, 2014. **11**(1): p. 32.

77. Muthuri, S.K., et al., *Associations Between Parental Perceptions of the Neighborhood Environment and Childhood Physical Activity: Results from ISCOLE-Kenya.* J Phys Act Health, 2016. **13**(3): p. 333-43.

78. Motl, R.W., et al., *Perceptions of physical and social environment variables and self-efficacy as correlates of self-reported physical activity among adolescent girls.* J Pediatr Psychol, 2007. **32**(1): p. 6-12.

79. McGinn, A.P., et al., *The association of perceived and objectively measured crime with physical activity: a cross-sectional analysis.* J Phys Act Health, 2008. **5**(1): p. 117-31.

80. McDonald, N.C., *Travel and the social environment: Evidence from Alameda County, California.* Transportation Research Part D: Transport and Environment, 2007. **12**(1): p. 53-63.

81. Machado-Rodrigues, A.M., et al., *Parental perceptions of neighborhood environments, BMI, and active behaviors in girls aged 7-9 years.* Am J Hum Biol, 2014. **26**(5): p. 670-5.

82. Loch, M.R., et al., *Relationship between social capital indicators and lifestyle in Brazilian adults.* Cadernos de Saúde Pública, 2015. **31**: p. 1636-1647.

83. Liao, Y., et al., *Perceived environmental factors associated with physical activity among normal-weight and overweight Japanese men.* Int J Environ Res Public Health, 2011. **8**(4): p. 931-43.

84. Li, Y., D. Kao, and T.Q. Dinh, *Correlates of neighborhood environment with walking among older Asian Americans.* J Aging Health, 2015. **27**(1): p. 17-34.

85. Li, M., et al., *Factors associated with adolescents' physical inactivity in Xi'an City, China.* Med Sci Sports Exerc, 2006. **38**(12): p. 2075-85.

86. Leslie, E., E. Cerin, and P. Kremer, *Perceived neighborhood environment and park use as mediators of the effect of area socio-economic status on walking behaviors.* J Phys Act Health, 2010. **7**(6): p. 802-10.

87. Lavin Fueyo, J., et al., *Neighborhood and family perceived environments associated with children's physical activity and body mass index.* Prev Med, 2016. **82**: p. 35-41.

88. Kurka, J.M., et al., *Patterns of neighborhood environment attributes in relation to children's physical activity.* Health Place, 2015. **34**: p. 164-70.

89. Kimbro, R.T., J. Brooks-Gunn, and S. McLanahan, *Young children in urban areas: links among neighborhood characteristics, weight status, outdoor play, and television watching.* Soc Sci Med, 2011. **72**(5): p. 668-76.

90. Kim, H.J. and K.M. Heinrich, *Built Environment Factors Influencing Walking to School Behaviors: A Comparison between a Small and Large US City.* Front Public Health, 2016. **4**: p. 77.

91. Kerr, Z., et al., *Changes in walking associated with perceived neighborhood safety and police-recorded crime: The multi-ethnic study of atherosclerosis.* Prev Med, 2015. **73**: p. 88-93.

92. Kerr, J., et al., *Exercise aids, neighborhood safety, and physical activity in adolescents and parents.* Med Sci Sports Exerc, 2008. **40**(7): p. 1244-8.

93. Jack, E. and G.R. McCormack, *The associations between objectively-determined and self-reported urban form characteristics and neighborhood-based walking in adults.* Int J Behav Nutr Phys Act, 2014. **11**: p. 71.

94. Inoue, S., et al., *Perceived neighborhood environment and walking for specific purposes among elderly Japanese.* J Epidemiol, 2011. **21**(6): p. 481-90.

95. Handy, S.L., X. Cao, and P.L. Mokhtarian, *The causal influence of neighborhood design on physical activity within the neighborhood: evidence from Northern California.* Am J Health Promot, 2008. **22**(5): p. 350-8.

96. Graziose, M.M., et al., *Association Between the Built Environment in School Neighborhoods With Physical Activity Among New York City Children, 2012.* Prev Chronic Dis, 2016. **13**: p. E110.

97. Glass, T.A., M.D. Rasmussen, and B.S. Schwartz, *Neighborhoods and obesity in older adults: the Baltimore Memory Study.* Am J Prev Med, 2006. **31**(6): p. 455-63.

98. Foster, S., et al., *Safe RESIDential Environments? A longitudinal analysis of the influence of crime-related safety on walking.* Int J Behav Nutr Phys Act, 2016. **13**: p. 22.

99. Evenson, K.R., et al., *Girls' perception of neighborhood factors on physical activity, sedentary behavior, and BMI.* Obesity (Silver Spring), 2007. **15**(2): p. 430-45.

100. Evenson, K.R., et al., *Associations of adult physical activity with perceived safety and police-recorded crime: the Multi-ethnic Study of Atherosclerosis.* Int J Behav Nutr Phys Act, 2012. **9**: p. 146.

101. Evenson, K.R., et al., *Girls' perception of physical environmental factors and transportation: reliability and association with physical activity and active transport to school.* Int J Behav Nutr Phys Act, 2006. **3**: p. 28.

102. Esteban-Cornejo, I., et al., *Parental and Adolescent Perceptions of Neighborhood Safety Related to Adolescents' Physical Activity in Their Neighborhood.* Res Q Exerc Sport, 2016. **87**(2): p. 191-9.

103. Eichinger, M., et al., *How are physical activity behaviors and cardiovascular risk factors associated with characteristics of the built and social residential environment?* PLoS One, 2015. **10**(6): p. e0126010.

104. D'Haese, S., et al., *The association between the parental perception of the physical neighborhood environment and children's location-specific physical activity.* BMC Public Health, 2015. **15**: p. 565.

105. Deweese, R.S., et al., *Neighborhood perceptions and active school commuting in low-income cities.* Am J Prev Med, 2013. **45**(4): p. 393-400.

106. Davison, K.K., et al., *Associations among social capital, parenting for active lifestyles, and youth physical activity in rural families living in upstate New York.* Soc Sci Med, 2012. **75**(8): p. 1488-96.

107. Datar, A., et al., *Neighborhood Environment and Children's Physical Activity and Body Mass Index: Evidence from Military Personnel Installation Assignments.* Child Obes, 2015.

108. Caspi, C.E., et al., *The social environment and walking behavior among low-income housing residents.* Soc Sci Med, 2013. **80**: p. 76-84.

109. Carver, A., A.F. Timperio, and D.A. Crawford, *Bicycles gathering dust rather than raising dust--Prevalence and predictors of cycling among Australian schoolchildren.* J Sci Med Sport, 2015. **18**(5): p. 540-4.

110. Carroll-Scott, A., et al., *Disentangling neighborhood contextual associations with child body mass index, diet, and physical activity: the role of built, socioeconomic, and social environments.* Soc Sci Med, 2013. **95**: p. 106-14.

111. Bringolf-Isler, B., et al., *Personal and environmental factors associated with active commuting to school in Switzerland.* Prev Med, 2008. **46**(1): p. 67-73.

112. Behanova, M., et al., *The effect of neighbourhood unemployment on health-risk behaviours in elderly differs between Slovak and Dutch cities.* Eur J Public Health, 2015. **25**(1): p. 108-14.

113. Ball, K., et al., *Love thy neighbour? Associations of social capital and crime with physical activity amongst women.* Soc Sci Med, 2010. **71**(4): p. 807-14.

114. Amorim, T.C., M.R. Azevedo, and P.C. Hallal, *Physical activity levels according to physical and social environmental factors in a sample of adults living in South Brazil.* J Phys Act Health, 2010. **7 Suppl 2**: p. S204-12.

115. Adlakha, D., et al., *Home and workplace built environment supports for physical activity.* Am J Prev Med, 2015. **48**(1): p. 104-7.

116. Wen, M. and X. Zhang, *Contextual effects of built and social environments of urban neighborhoods on exercise: a multilevel study in Chicago.* Am J Health Promot, 2009. **23**(4): p. 247-54.

117. Ueshima, K., et al., *Does social capital promote physical activity? A population-based study in Japan.* PLoS One, 2010. **5**(8): p. e12135.

118. Timperio, A., J. Veitch, and A. Carver, *Safety in numbers: Does perceived safety mediate associations between the neighborhood social environment and physical activity among women living in disadvantaged neighborhoods?* Prev Med, 2015.

119. Sugiyama, T., et al., *Neighborhood environmental attributes and adults' maintenance of regular walking.* Med Sci Sports Exerc, 2015. **47**(6): p. 1204-10.

120. Solomon, E., et al., *Personal, social, and environmental correlates of physical activity in adults living in rural south-west England: a cross-sectional analysis.* Int J Behav Nutr Phys Act, 2013. **10**: p. 129.

121. Quon, E.C. and J.J. McGrath, *Community, Family, and Subjective Socioeconomic Status: Relative Status and Adolescent Health.* Health Psychol, 2014.

122. Piro, F.N., O. Noss, and B. Claussen, *Physical activity among elderly people in a city population: the influence of neighbourhood level violence and self perceived safety.* J Epidemiol Community Health, 2006. **60**(7): p. 626-32.

123. Pelclova, J., K. Fromel, and R. Cuberek, *Gender-specific associations between perceived neighbourhood walkability and meeting walking recommendations when walking for transport and recreation for Czech inhabitants over 50 years of age.* Int J Environ Res Public Health, 2014. **11**(1): p. 527-36.

124. Mendes de Leon, C.F., et al., *Neighborhood social cohesion and disorder in relation to walking in community-dwelling older adults: a multilevel analysis.* J Aging Health, 2009. **21**(1): p. 155-71.

125. Mason, P., A. Kearns, and M. Livingston, *"Safe Going": the influence of crime rates and perceived crime and safety on walking in deprived neighbourhoods.* Soc Sci Med, 2013. **91**: p. 15-24.

126. Lee, R.E., C. Cubbin, and M. Winkleby, *Contribution of neighbourhood socioeconomic status and physical activity resources to physical activity among women.* J Epidemiol Community Health, 2007. **61**(10): p. 882-90.

127. Kamphuis, C.B., et al., *Socioeconomic status, environmental and individual factors, and sports participation.* Med Sci Sports Exerc, 2008. **40**(1): p. 71-81.

128. Heitzler, C.D., et al., *Correlates of physical activity in a national sample of children aged 9-13 years.* Prev Med, 2006. **42**(4): p. 254-60.

129. Gao, J., *Association between social and built environments and leisure-time physical activity among Chinese older adults--a multilevel analysis.* BMC Public Health, 2015. **15**: p. 1317-28.

130. Foster, S., et al., *Does walkable neighbourhood design influence the association between objective crime and walking?* Int J Behav Nutr Phys Act, 2014. **11**: p. 100.

131. Forsyth, A., et al., *Perceived and Police-Reported Neighborhood Crime: Linkages to Adolescent Activity Behaviors and Weight Status.* J Adolesc Health, 2015. **57**(2): p. 222-8.

132. Chen, T.A., et al., *Features of perceived neighborhood environment associated with daily walking time or habitual exercise: differences across gender, age, and employment status in a community-dwelling population of Japan.* Environ Health Prev Med, 2013. **18**(5): p. 368-76.

133. Carlson, S.A., et al., *How reported usefulness modifies the association between neighborhood supports and walking behavior.* Prev Med, 2016. **91**: p. 76-81.

134. Borrell, L.N., et al., *Racial discrimination, racial/ethnic segregation, and health behaviors in the CARDIA study.* Ethn Health, 2013. **18**(3): p. 227-43.

135. Boone-Heinonen, J. and P. Gordon-Larsen, *Life stage and sex specificity in relationships between the built and socioeconomic environments and physical activity.* J Epidemiol Community Health, 2011. **65**(10): p. 847-52.

136. Boclin Kde, L., E. Faerstein, and A.C. Leon, *Neighborhood contextual characteristics and leisure-time physical activity: Pro-Saude Study.* Rev Saude Publica, 2014. **48**(2): p. 249-57.

137. Beenackers, M.A., et al., *Why some walk and others don't: exploring interactions of perceived safety and social neighborhood factors with psychosocial cognitions.* Health Educ Res, 2013. **28**(2): p. 220-33.

138. Andrade, A.C., et al., *Social context of neighborhood and socioeconomic status on leisure-time physical activity in a Brazilian urban center: The BH Health Study.* Cad Saude Publica, 2015. **31 Suppl 1**: p. 136-47.

139. Alves, L., et al., *Association between neighborhood deprivation and fruits and vegetables consumption and leisure-time physical activity: a cross-sectional multilevel analysis.* BMC Public Health, 2013. **13**: p. 1103.

140. Adams, R.J., et al., *Effects of area deprivation on health risks and outcomes: a multilevel, cross-sectional, Australian population study.* Int J Public Health, 2009. **54**(3): p. 183-92.

141. Aarts, M.J., et al., *Associations between environmental characteristics and active commuting to school among children: a cross-sectional study.* Int J Behav Med, 2013. **20**(4): p. 538-55.

142. Aarts, M.J., et al., *Environmental determinants of outdoor play in children: a large-scale cross-sectional study.* Am J Prev Med, 2010. **39**(3): p. 212-9.

143. Astell-Burt, T., X. Feng, and G.S. Kolt, *Identification of the impact of crime on physical activity depends upon neighbourhood scale: Multilevel evidence from 203,883 Australians.* Health Place, 2015. **31**: p. 120-3.

144. Beets, M.W. and J.T. Foley, *Association of father involvement and neighborhood quality with kindergartners' physical activity: a multilevel structural equation model.* Am J Health Promot, 2008. **22**(3): p. 195-203.

145. Bolivar, J., et al., *The influence of individual, social and physical environment factors on physical activity in the adult population in Andalusia, Spain.* Int J Environ Res Public Health, 2010. **7**(1): p. 60-77.

146. Datar, A., N. Nicosia, and V. Shier, *Parent perceptions of neighborhood safety and children's physical activity, sedentary behavior, and obesity: evidence from a national longitudinal study.* Am J Epidemiol, 2013. **177**(10): p. 1065-73.

147. Ding, D., et al., *Perceived neighborhood environment and physical activity in 11 countries: do associations differ by country?* Int J Behav Nutr Phys Act, 2013. **10**: p. 57.

148. Doyle, S., et al., *Active Community Environments and Health: The Relationship of Walkable and Safe Communities to Individual Health.* Journal of the American Planning Association, 2006. **72**(1): p. 19-31.

149. Dragano, N., et al., *Neighbourhood socioeconomic status and cardiovascular risk factors: a multilevel analysis of nine cities in the Czech Republic and Germany.* BMC Public Health, 2007. **7**: p. 255.

150. Duke, N.N., I.W. Borowsky, and S.L. Pettingell, *Parent perceptions of neighborhood: relationships with US youth physical activity and weight status.* Matern Child Health J, 2012. **16**(1): p. 149-57.

151. Echeverria, S., et al., *Associations of neighborhood problems and neighborhood social cohesion with mental health and health behaviors: the Multi-Ethnic Study of Atherosclerosis.* Health Place, 2008. **14**(4): p. 853-65.

152. Garrett, N., P.J. Schluter, and G. Schofield, *Physical activity profiles and perceived environmental determinants in New Zealand: a national cross-sectional study.* J Phys Act Health, 2012. **9**(3): p. 367-77.

153. Gomes, C.S., et al., *Physical and Social Environment Are Associated to Leisure Time Physical Activity in Adults of a Brazilian City: A Cross-Sectional Study.* PLoS One, 2016. **11**(2): p. e0150017.

154. Harrison, R.A., I. Gemmell, and R.F. Heller, *The population effect of crime and neighbourhood on physical activity: an analysis of 15,461 adults.* J Epidemiol Community Health, 2007. **61**(1): p. 34-9.

155. Janke, K., C. Propper, and M.A. Shields, *Assaults, murders and walkers: The impact of violent crime on physical activity.* J Health Econ, 2016. **47**: p. 34-49.

156. Jones, A., M. Hillsdon, and E. Coombes, *Greenspace access, use, and physical activity: understanding the effects of area deprivation.* Prev Med, 2009. **49**(6): p. 500-5.

157. Karusisi, N., et al., *Multiple dimensions of residential environments, neighborhood experiences, and jogging behavior in the RECORD Study.* Prev Med, 2012. **55**(1): p. 50-5.

158. Kramer, D., et al., *Neighbourhood safety and leisure-time physical activity among Dutch adults: a multilevel perspective.* Int J Behav Nutr Phys Act, 2013. **10**: p. 11.

159. Kramer, D., et al., *Social neighborhood environment and sports participation among Dutch adults: does sports location matter?* Scand J Med Sci Sports, 2015. **25**(2): p. 273-9.

160. Logstein, B., A. Blekesaune, and R. Almas, *Physical activity among Norwegian adolescents--a multilevel analysis of how place of residence is associated with health behaviour: the Young-HUNT study.* Int J Equity Health, 2013. **12**: p. 56.

161. Mackenbach, J.D., et al., *Exploring why residents of socioeconomically deprived neighbourhoods have less favourable perceptions of their neighbourhood environment than residents of wealthy neighbourhoods.* Obes Rev, 2016. **17 Suppl 1**: p. 42-52.

162. Mackenbach, J.D., et al., *Neighbourhood social capital: measurement issues and associations with health outcomes.* Obes Rev, 2016. **17 Suppl 1**: p. 96-107.

163. Macniven, R., et al., *Physical activity, healthy lifestyle behaviors, neighborhood environment characteristics and social support among Australian Aboriginal and non-Aboriginal adults.* Prev Med Rep, 2016. **3**: p. 203-10.

164. Mohnen, S.M., et al., *Health-related behavior as a mechanism behind the relationship between neighborhood social capital and individual health--a multilevel analysis.* BMC Public Health, 2012. **12**: p. 116.

165. Reis, R.S., et al., *Assessing participation in community-based physical activity programs in Brazil.* Med Sci Sports Exerc, 2014. **46**(1): p. 92-8.

166. Rind, E., et al., *Are income-related differences in active travel associated with physical environmental characteristics? A multi-level ecological approach.* Int J Behav Nutr Phys Act, 2015. **12**: p. 73.

167. Samuel, L.J., et al., *Social engagement and chronic disease risk behaviors: The Multi-Ethnic Study of Atherosclerosis.* Prev Med, 2015. **71**: p. 61-6.

168. Santana, P., R. Santos, and H. Nogueira, *The link between local environment and obesity: a multilevel analysis in the Lisbon Metropolitan Area, Portugal.* Soc Sci Med, 2009. **68**(4): p. 601-9.

169. Santos, M.S., et al., *Socio-demographic and perceived environmental correlates of walking in Portuguese adults--a multilevel analysis.* Health Place, 2009. **15**(4): p. 1094-9.

170. Santos, R., et al., *Physical activity and perceived environmental attributes in a sample of Portuguese adults: results from the Azorean Physical Activity and Health study.* Prev Med, 2008. **47**(1): p. 83-8.

171. Shenassa, E.D., A. Liebhaber, and A. Ezeamama, *Perceived safety of area of residence and exercise: a pan-European study.* Am J Epidemiol, 2006. **163**(11): p. 1012-7.

172. Singh, G.K., et al., *Independent and joint effects of socioeconomic, behavioral, and neighborhood characteristics on physical inactivity and activity levels among US children and adolescents.* J Community Health, 2008. **33**(4): p. 206-16.

173. Singh, G.K., et al., *Prevalence and correlates of state and regional disparities in vigorous physical activity levels among US children and adolescents.* J Phys Act Health, 2009. **6**(1): p. 73-87.

174. Stimpson, J.P., et al., *Neighborhood Deprivation is associated with lower levels of serum carotenoids among adults participating in the Third National Health and Nutrition Examination Survey.* J Am Diet Assoc, 2007. **107**(11): p. 1895-902.

175. Troped, P.J., et al., *Perceived built environment and physical activity in U.S. women by sprawl and region.* Am J Prev Med, 2011. **41**(5): p. 473-9.

176. Turrell, G., et al., *Neighborhood disadvantage and physical activity: baseline results from the HABITAT multilevel longitudinal study.* Ann Epidemiol, 2010. **20**(3): p. 171-81.

177. Vamos, C.A., et al., *Community Level Predictors of Physical Activity Among Women in the Preconception Period.* Matern Child Health J, 2015.

178. Van Cauwenberg, J., et al., *Relationships between the perceived neighborhood social environment and walking for transportation among older adults.* Soc Sci Med, 2014. **104**: p. 23-30.

179. Van Dyck, D., et al., *Moderating effects of age, gender and education on the associations of perceived neighborhood environment attributes with accelerometer-based physical activity: The IPEN adult study.* Health Place, 2015. **36**: p. 65-73.

180. Wen, M., N.R. Kandula, and D.S. Lauderdale, *Walking for transportation or leisure: what difference does the neighborhood make?* J Gen Intern Med, 2007. **22**(12): p. 1674-80.

181. Kelly, C.M., et al., *Walking to Work: The Roles of Neighborhood Walkability and Socioeconomic Deprivation.* J Phys Act Health, 2013.
