# Supplementary material for: The neighborhood social environment and physical activity: a systematic scoping review
Source: Int J Behav Nutr Phys Act. 2019 Dec 9;16:124. doi: 10.1186/s12966-019-0873-7 (PMC6902518; doi:10.1186/s12966-019-0873-7)
Supplement: Supplementary file 5 — Additional file 5: Table S3. Summary of social environmental constructs and measurements used in 181 published studies on physical activity. [file 12966_2019_873_MOESM5_ESM.docx]

| **Table S3: Summary of social environmental constructs and measurements used in 181 published studies on physical activity.** | | | | | | |
| --- | --- | --- | --- | --- | --- | --- |
| **Social Environment (SE) Dimensions** | | **# of studies that included the SE construct** | **# of times the SE construct was measured^2^** | **# of times a unique measurement was used^3^** | **N(%) of times measured at aggregate level^4^** | **N(%) of times referred to as the SE^5^** |
|  | **SE Constructs^1^** |  |  |  |  |  |
| ***Social Inequalities*** | | | | | | |
| **Economic and Social Disadvantage** | | | | | | |
|  | **Total** | **55[1-55]** | **66** | **42** | **65(98)** | **8(12)** |
|  | Affluence | 1 [1] | 1 | 1 | 0(0) | 0(0) |
|  | Education* | 4 [2-5] | 4 | 1 | 4(100) | 0(0) |
|  | Income | 5 [3, 4, 6-8] | 5 | 3 | 5(100) | 0(0) |
|  | Income Distribution* | 1 [9] | 1 | 1 | 1 (100) | 0(0) |
|  | Income Inequality | 2[10, 11] | 2 | 2 | 2(100) | 1(50) |
|  | Poverty | 7 [2, 4, 10, 12-15] | 7 | 1 | 7(100) | 0(0) |
|  | Socioeconomic Status (SES) | 21 [5, 11, 16-34] | 22 | 16 | 22(100) | 4(18) |
|  | Deprivation | 17[1, 35-50] | 18 | 13 | 18(100) | 2(11) |
|  | Disadvantage | 4[51-54] | 4 | 2 | 4(100) | 0(0) |
|  | Social Development* | 1[9] | 1 | 1 | 1(100) | 0(0) |
|  | Structural Characteristics* | 1[55] | 1 | 1 | 1(100) | 1(100) |
| **Discrimination and Segregation** | | | | | | |
|  | **Total** | **3 [2, 34, 56]** | **3** | **3** | **2(67)** | **0(0)** |
|  | Racism | 1 [56] | 1 | 1 | 0(0) | 0(0) |
|  | Racial Segregation | 2 [2, 34] | 2 | 2 | 2(100) | 0(0) |
| ***Neighborhood & Community Characteristics*** | | | | | | |
| **Crime and Safety** | | | | | | |
|  | **Total** | **133[5, 6, 8, 10, 13, 14, 17, 20-22, 25-30, 32, 33, 38-41, 43, 44, 50, 51, 53, 55, 57-161]** | **179** | **31** | **38(21)** | **19(11)** |
|  | Neighborhood Danger* | 1[39] | 1 | 1 | 1 (100) | 1(100) |
|  | Crime | 48 [5, 6, 8, 10, 20, 21, 25, 26, 50, 51, 55, 57-93] | 54 | 12 | 28(52) | 6(13) |
|  | Safety | 95 [6, 10, 13, 14, 17, 27-30, 32, 33, 38, 40, 41, 43, 44, 50, 53, 57, 61, 63, 65, 68-70, 73, 78, 80, 87, 93-158] | 115 | 9 | 7(6) | 9(8) |
|  | Stranger Danger* | 2[22, 77] | 2 | 2 | 0(0) | 2(100) |
|  | Violence* | 4[79, 91, 108, 137] | 4 | 4 | 1(25) | 1(25) |
|  | Risk* | 1[159] | 1 | 1 | 0(0) | 0(0) |
|  | Environmental Barriers* | 2{Garrett, 2012 #1453;Jilcott Pitts, 2015 #109} | 2 | 2 | 1(50) | 0(0) |
| **Social Cohesion and Social Capital** | | | | | | |
|  | **Total** | **47 [3, 7, 10, 12, 14, 19, 28-30, 32, 39, 40, 60, 63, 70, 77, 80, 82, 91, 93, 96-98, 104, 107, 108, 117, 120, 121, 125, 128, 134, 144, 154, 162-174]** | **53** | **40** | **15(28)** | **23(43)** |
|  | Social Capital | 16 [7, 10, 32, 40, 60, 107, 121, 128, 134, 144, 162-167] | 16 | 12 | 5(31.3) | 5(31) |
|  | Social Cohesion | 26 [3, 14, 19, 28-30, 39, 60, 70, 82, 91, 93, 96-98, 104, 117, 120, 125, 154, 168-173] | 26 | 19 | 6(24) | 16(61.5) |
|  | Collective Insecurity* | 1 [3] | 1 | 1 | 1(100) | 0(0) |
|  | Trust* | 3 [60, 63, 174] | 3 | 3 | 1(33.3) | 0 (0) |
|  | Collective Efficacy | 3 [12, 77, 108] | 3 | 1 | 0(0) | 2(66.7) |
|  | Hostility and Distrust* | 1 [3] | 1 | 1 | 1(100) | 0(0) |
|  | Psychosocial Aspects* | 1 [80] | 1 | 1 | 0(0) | 0(0) |
|  | Norms of Reciprocity* | 1[60] | 1 | 1 | 1(100) | 0(0) |
|  | Social Control | 1 [96] | 1 | 1 | 0(0) | 0(0) |
| **Sense of Place/Belonging** | | | | | | |
|  | **Total** | **8 [15, 39, 50, 96, 98, 116, 117, 175]** | **8** | **8** | **2(25)** | **4(50)** |
|  | Place Attachment* | 2[96, 175] | 2 | 2 | 0(0) | 1(50) |
|  | Sense of Community* | 1 [116] | 1 | 1 | 0(0) | 0(0) |
|  | Territoriality* | 1[15] | 1 | 1 | 1(100) | 1(100) |
|  | Feeling at Home* | 2 [98, 117] | 2 | 2 | 0(0) | 1(50) |
|  | Social fragmentation* | 1[39] | 1 | 1 | 1(100) | 1(100) |
|  | Belonging* | 1[50] | 1 | 1 | 0(0) | 0(0) |
| **Disorder and Incivilities** | | | | | | |
|  | **Total** | **15[4, 12, 15, 19, 29, 30, 32, 39, 51, 61, 78, 80, 117, 168, 172]** | **20** | **15** | **11(55)** | **11(55)** |
|  | Incivilities* | 2 [4, 51] | 2 | 2 | 2(100) | 0(0) |
|  | Disorder | 7 [12, 15, 32, 39, 61, 78, 172] | 9 | 7 | 8(89) | 4(44) |
|  | Dog Dirt or Waste/Litter* | 2[29, 30] | 2 | 1 | 0(0) | 2(100) |
|  | Unoccupied Housing* | 2[29, 30] | 2 | 1 | 0(0) | 2(100) |
|  | Problems* | 3[19, 80, 168] | 3 | 2 | 0(0) | 1(33.3) |
|  | Territoriality* | 1[15] | 1 | 1 | 1(100) | 1(100) |
|  | Social Disorganization* | 1[117] | 1 | 1 | 0(0) | 1(100) |
| ***Social Interactions in the Neighborhood*** | | | | | | |
| **Social Relationships and Norms** | | | | | | |
|  | **Total** | **22[3, 22, 23, 30, 37, 51, 60, 65, 67, 75, 91, 96, 98, 113, 117, 133, 146, 170, 171, 175-177]** | **26** | **25** | **4(15)** | **13(50)** |
|  | Social Norms | 2[91, 146] | 3 | 3 | 0(0) | 1(33.3) |
|  | Social Networks | 2 [98, 117] | 2 | 1 | 0(0) | 1(50) |
|  | Social Contacts* | 1 [30] | 1 | 1 | 0(0) | 1(100) |
|  | Neighborhood Experiences* | 1 [3] | 1 | 1 | 0(0) | 0(0) |
|  | Social Connectedness | 2 [60, 75] | 2 | 2 | 0(0) | 0(0) |
|  | Social Support | 5 [51, 96, 133, 170, 176] | 5 | 5 | 0(0) | 2(40) |
|  | Social Ties* | 1 [65] | 1 | 1 | 0(0) | 1(100) |
|  | Social Participation* | 3[37, 60, 171] | 3 | 3 | 2(67) | 1(33) |
|  | Interpersonal Relationships* | 1[175] | 1 | 1 | 0(0) | 1(100) |
|  | Social Interaction* | 2 [3, 67] | 2 | 2 | 0(0) | 0(0) |
|  | Socializing* | 1[113] | 1 | 1 | 0(0) | 1(100) |
|  | Social Contacts* | 1[30] | 1 | 1 | 0(0) | 1(100) |
|  | Social Relations* | 1[177] | 1 | 1 | 1(100) | 1(100) |
|  | Other Children in Neighborhood* | 2[22, 23] | 2 | 2 | 1(50.0) | 2(100) |
| **Civic Participation/Engagement** | | | | | | |
|  | **Total** | **2 [14, 175]** | **2** | **2** | **1(50)** | **1(50)** |
|  | Civic Participation | 1 [14] | 1 | 1 | 1(100) | 0(0) |
|  | Community Engagement* | 1[175] | 1 | 1 | 0(0) | 1(100) |
| **Social Environment** | | | | | | |
|  | Social Environment | 16[83, 101, 115, 118, 123, 127, 130-132, 141, 142, 155, 178-181] | 16 | 10 | 2(13) | 16(100) |
| ^1^ SE constructs with a * were identified inductively, which means these constructs were not identified as the neighborhood social environment prior to this review (deductive code).  ^2^ This column is the number of times the construct was measured. This number may be greater than the number of studies that included the SE construct, as a single study may have included the same SE construct measured in two different ways. This commonly occurred when authors measured a SE construct at both the individual and aggregate level in the same study.  ^3^This column reflects the number of unique ways each SE construct was measured across all articles. The total for each domain is the sum of all constructs within the domain. Therefore, if two constructs within a domain were measured using the same method the total may be inflated. This would occur if authors used the same measurement but called it different things, as constructs reflect how authors referred to the SE.  ^4^% of times measured at aggregate level= # of times measured at the aggregate-level/# of times measured across all articles.  ^5^% of times referred to as the SE= # of times the author referred to the construct as the SE/# of times measured across all articles. | | | | | | |

1. Glass, T.A., M.D. Rasmussen, and B.S. Schwartz, *Neighborhoods and obesity in older adults: the Baltimore Memory Study.* Am J Prev Med, 2006. **31**(6): p. 455-63.

2. Armstrong-Brown, J., et al., *Redefining Racial Residential Segregation and its Association With Physical Activity Among African Americans 50 Years and Older: A Mixed Methods Approach.* J Aging Phys Act, 2014.

3. Karusisi, N., et al., *Multiple dimensions of residential environments, neighborhood experiences, and jogging behavior in the RECORD Study.* Prev Med, 2012. **55**(1): p. 50-5.

4. Rossen, L.M., et al., *Neighborhood incivilities, perceived neighborhood safety, and walking to school among urban-dwelling children.* J Phys Act Health, 2011. **8**(2): p. 262-71.

5. Vamos, C.A., et al., *Community Level Predictors of Physical Activity Among Women in the Preconception Period.* Matern Child Health J, 2015.

6. Kerr, J., et al., *Active commuting to school: Associations with environment and parental concerns.* Med Sci Sports Exerc, 2006. **38**(4): p. 787-94.

7. Mohnen, S.M., et al., *Health-related behavior as a mechanism behind the relationship between neighborhood social capital and individual health--a multilevel analysis.* BMC Public Health, 2012. **12**: p. 116.

8. Gomes, C.S., et al., *Physical and Social Environment Are Associated to Leisure Time Physical Activity in Adults of a Brazilian City: A Cross-Sectional Study.* PLoS One, 2016. **11**(2): p. e0150017.

9. Boclin Kde, L., E. Faerstein, and A.C. Leon, *Neighborhood contextual characteristics and leisure-time physical activity: Pro-Saude Study.* Rev Saude Publica, 2014. **48**(2): p. 249-57.

10. Singh, G.K., et al., *Prevalence and correlates of state and regional disparities in vigorous physical activity levels among US children and adolescents.* J Phys Act Health, 2009. **6**(1): p. 73-87.

11. Quon, E.C. and J.J. McGrath, *Community, Family, and Subjective Socioeconomic Status: Relative Status and Adolescent Health.* Health Psychol, 2014.

12. Kimbro, R.T., J. Brooks-Gunn, and S. McLanahan, *Young children in urban areas: links among neighborhood characteristics, weight status, outdoor play, and television watching.* Soc Sci Med, 2011. **72**(5): p. 668-76.

13. Lovasi, G.S., et al., *Is the environment near home and school associated with physical activity and adiposity of urban preschool children?* J Urban Health, 2011. **88**(6): p. 1143-57.

14. Osypuk, T.L., et al., *Are immigrant enclaves healthy places to live? The Multi-ethnic Study of Atherosclerosis.* Soc Sci Med, 2009. **69**(1): p. 110-20.

15. Schulz, A., et al., *Independent and joint associations between multiple measures of the built and social environment and physical activity in a multi-ethnic urban community.* J Urban Health, 2013. **90**(5): p. 872-87.

16. Lee, R.E., C. Cubbin, and M. Winkleby, *Contribution of neighbourhood socioeconomic status and physical activity resources to physical activity among women.* J Epidemiol Community Health, 2007. **61**(10): p. 882-90.

17. Leslie, E., E. Cerin, and P. Kremer, *Perceived neighborhood environment and park use as mediators of the effect of area socio-economic status on walking behaviors.* J Phys Act Health, 2010. **7**(6): p. 802-10.

18. Dragano, N., et al., *Neighbourhood socioeconomic status and cardiovascular risk factors: a multilevel analysis of nine cities in the Czech Republic and Germany.* BMC Public Health, 2007. **7**: p. 255.

19. Echeverria, S., et al., *Associations of neighborhood problems and neighborhood social cohesion with mental health and health behaviors: the Multi-Ethnic Study of Atherosclerosis.* Health Place, 2008. **14**(4): p. 853-65.

20. McGinn, A.P., et al., *The association of perceived and objectively measured crime with physical activity: a cross-sectional analysis.* J Phys Act Health, 2008. **5**(1): p. 117-31.

21. Ribeiro, A.I., et al., *Distance to parks and non-residential destinations influences physical activity of older people, but crime doesn't: a cross-sectional study in a southern European city.* BMC Public Health, 2015. **15**: p. 593.

22. Timperio, A., et al., *Personal, family, social, and environmental correlates of active commuting to school.* Am J Prev Med, 2006. **30**(1): p. 45-51.

23. van Loon, J., et al., *Youth physical activity and the neighbourhood environment: examining correlates and the role of neighbourhood definition.* Soc Sci Med, 2014. **104**: p. 107-15.

24. Voorhees, C.C., et al., *Neighborhood socioeconomic status and non school physical activity and body mass index in adolescent girls.* J Phys Act Health, 2009. **6**(6): p. 731-40.

25. Foster, S., et al., *Does walkable neighbourhood design influence the association between objective crime and walking?* Int J Behav Nutr Phys Act, 2014. **11**: p. 100.

26. Kremers, S.P., et al., *Associations between safety from crime, cycling, and obesity in a Dutch elderly population: results from the Longitudinal Aging Study Amsterdam.* J Environ Public Health, 2012. **2012**: p. 127857.

27. Van Holle, V., et al., *The Association between Belgian Older Adults' Physical Functioning and Physical Activity: What Is the Moderating Role of the Physical Environment?* PLoS One, 2016. **11**(2): p. e0148398.

28. Wen, M., N.R. Kandula, and D.S. Lauderdale, *Walking for transportation or leisure: what difference does the neighborhood make?* J Gen Intern Med, 2007. **22**(12): p. 1674-80.

29. Aarts, M.J., et al., *Associations between environmental characteristics and active commuting to school among children: a cross-sectional study.* Int J Behav Med, 2013. **20**(4): p. 538-55.

30. Aarts, M.J., et al., *Environmental determinants of outdoor play in children: a large-scale cross-sectional study.* Am J Prev Med, 2010. **39**(3): p. 212-9.

31. Bolivar, J., et al., *The influence of individual, social and physical environment factors on physical activity in the adult population in Andalusia, Spain.* Int J Environ Res Public Health, 2010. **7**(1): p. 60-77.

32. Caspi, C.E., et al., *The social environment and walking behavior among low-income housing residents.* Soc Sci Med, 2013. **80**: p. 76-84.

33. Evenson, K.R., et al., *Girls' perception of neighborhood factors on physical activity, sedentary behavior, and BMI.* Obesity (Silver Spring), 2007. **15**(2): p. 430-45.

34. Borrell, L.N., et al., *Racial discrimination, racial/ethnic segregation, and health behaviors in the CARDIA study.* Ethn Health, 2013. **18**(3): p. 227-43.

35. Kelly, C.M., et al., *Walking to Work: The Roles of Neighborhood Walkability and Socioeconomic Deprivation.* J Phys Act Health, 2013.

36. Annear, M.J., G. Cushman, and B. Gidlow, *Leisure time physical activity differences among older adults from diverse socioeconomic neighborhoods.* Health Place, 2009. **15**(2): p. 482-90.

37. Logstein, B., A. Blekesaune, and R. Almas, *Physical activity among Norwegian adolescents--a multilevel analysis of how place of residence is associated with health behaviour: the Young-HUNT study.* Int J Equity Health, 2013. **12**: p. 56.

38. Noonan, R.J., et al., *Cross-sectional associations between high-deprivation home and neighbourhood environments, and health-related variables among Liverpool children.* BMJ Open, 2016. **6**(1): p. e008693.

39. Pabayo, R., et al., *The relationship between neighborhood socioeconomic characteristics and physical inactivity among adolescents living in Boston, Massachusetts.* Am J Public Health, 2014. **104**(11): p. e142-9.

40. Santana, P., R. Santos, and H. Nogueira, *The link between local environment and obesity: a multilevel analysis in the Lisbon Metropolitan Area, Portugal.* Soc Sci Med, 2009. **68**(4): p. 601-9.

41. Solomon, E., et al., *Personal, social, and environmental correlates of physical activity in adults living in rural south-west England: a cross-sectional analysis.* Int J Behav Nutr Phys Act, 2013. **10**: p. 129.

42. Stimpson, J.P., et al., *Neighborhood Deprivation is associated with lower levels of serum carotenoids among adults participating in the Third National Health and Nutrition Examination Survey.* J Am Diet Assoc, 2007. **107**(11): p. 1895-902.

43. Zandieh, R., et al., *Older Adults' Outdoor Walking: Inequalities in Neighbourhood Safety, Pedestrian Infrastructure and Aesthetics.* Int J Environ Res Public Health, 2016. **13**(12).

44. Jones, A., M. Hillsdon, and E. Coombes, *Greenspace access, use, and physical activity: understanding the effects of area deprivation.* Prev Med, 2009. **49**(6): p. 500-5.

45. Rind, E., et al., *Are income-related differences in active travel associated with physical environmental characteristics? A multi-level ecological approach.* Int J Behav Nutr Phys Act, 2015. **12**: p. 73.

46. Behanova, M., et al., *The effect of neighbourhood unemployment on health-risk behaviours in elderly differs between Slovak and Dutch cities.* Eur J Public Health, 2015. **25**(1): p. 108-14.

47. Alves, L., et al., *Association between neighborhood deprivation and fruits and vegetables consumption and leisure-time physical activity: a cross-sectional multilevel analysis.* BMC Public Health, 2013. **13**: p. 1103.

48. Boone-Heinonen, J. and P. Gordon-Larsen, *Life stage and sex specificity in relationships between the built and socioeconomic environments and physical activity.* J Epidemiol Community Health, 2011. **65**(10): p. 847-52.

49. Fox, K.R., et al., *Neighbourhood deprivation and physical activity in UK older adults.* Health Place, 2011. **17**(2): p. 633-40.

50. Harrison, R.A., I. Gemmell, and R.F. Heller, *The population effect of crime and neighbourhood on physical activity: an analysis of 15,461 adults.* J Epidemiol Community Health, 2007. **61**(1): p. 34-9.

51. Soltero, E.G., et al., *Does social support mediate the relationship among neighborhood disadvantage, incivilities, crime and physical activity?* Prev Med, 2015. **72**: p. 44-9.

52. Turrell, G., et al., *Neighborhood disadvantage and physical activity: baseline results from the HABITAT multilevel longitudinal study.* Ann Epidemiol, 2010. **20**(3): p. 171-81.

53. Sugiyama, T., et al., *Do Relationships Between Environmental Attributes and Recreational Walking Vary According to Area-Level Socioeconomic Status?* J Urban Health, 2015.

54. Adams, R.J., et al., *Effects of area deprivation on health risks and outcomes: a multilevel, cross-sectional, Australian population study.* Int J Public Health, 2009. **54**(3): p. 183-92.

55. McDonald, N.C., *Travel and the social environment: Evidence from Alameda County, California.* Transportation Research Part D: Transport and Environment, 2007. **12**(1): p. 53-63.

56. Edwards, M. and G. Cunningham, *Examining the associations of perceived community racism with self-reported physical activity levels and health among older racial minority adults.* J Phys Act Health, 2013. **10**(7): p. 932-9.

57. Oh, A.Y., et al., *Effects of perceived and objective neighborhood crime on walking frequency among midlife African American women in a home-based walking intervention.* J Phys Act Health, 2010. **7**(4): p. 432-41.

58. Adlakha, D., et al., *Home and workplace built environment supports for physical activity.* Am J Prev Med, 2015. **48**(1): p. 104-7.

59. Astell-Burt, T., X. Feng, and G.S. Kolt, *Identification of the impact of crime on physical activity depends upon neighbourhood scale: Multilevel evidence from 203,883 Australians.* Health Place, 2015. **31**: p. 120-3.

60. Ball, K., et al., *Love thy neighbour? Associations of social capital and crime with physical activity amongst women.* Soc Sci Med, 2010. **71**(4): p. 807-14.

61. Beets, M.W. and J.T. Foley, *Association of father involvement and neighborhood quality with kindergartners' physical activity: a multilevel structural equation model.* Am J Health Promot, 2008. **22**(3): p. 195-203.

62. Broyles, S.T., et al., *The Influence of Neighborhood Crime on Increases in Physical Activity during a Pilot Physical Activity Intervention in Children.* J Urban Health, 2016. **93**(2): p. 271-8.

63. Bungum, T.J., et al., *Perceived environmental physical activity correlates among Asian Pacific Islander Americans.* J Phys Act Health, 2012. **9**(8): p. 1098-104.

64. Carlson, S.A., et al., *How reported usefulness modifies the association between neighborhood supports and walking behavior.* Prev Med, 2016. **91**: p. 76-81.

65. Carroll-Scott, A., et al., *Disentangling neighborhood contextual associations with child body mass index, diet, and physical activity: the role of built, socioeconomic, and social environments.* Soc Sci Med, 2013. **95**: p. 106-14.

66. Doyle, S., et al., *Active Community Environments and Health: The Relationship of Walkable and Safe Communities to Individual Health.* Journal of the American Planning Association, 2006. **72**(1): p. 19-31.

67. Durand, C.P., et al., *Does community type moderate the relationship between parent perceptions of the neighborhood and physical activity in children?* Am J Health Promot, 2012. **26**(6): p. 371-80.

68. Evenson, K.R., et al., *Associations of adult physical activity with perceived safety and police-recorded crime: the Multi-ethnic Study of Atherosclerosis.* Int J Behav Nutr Phys Act, 2012. **9**: p. 146.

69. Forsyth, A., et al., *Perceived and Police-Reported Neighborhood Crime: Linkages to Adolescent Activity Behaviors and Weight Status.* J Adolesc Health, 2015. **57**(2): p. 222-8.

70. Foster, S., et al., *Safe RESIDential Environments? A longitudinal analysis of the influence of crime-related safety on walking.* Int J Behav Nutr Phys Act, 2016. **13**: p. 22.

71. Graziose, M.M., et al., *Association Between the Built Environment in School Neighborhoods With Physical Activity Among New York City Children, 2012.* Prev Chronic Dis, 2016. **13**: p. E110.

72. Jago, R., T. Baranowski, and M. Harris, *Relationships Between GIS Environmental Features and Adolescent Male Physical Activity: GIS Coding Differences.* J Phys Act Health, 2006. **3**(2): p. 230-242.

73. Jago, R., T. Baranowski, and J.C. Baranowski, *Observed, GIS, and self-reported environmental features and adolescent physical activity.* Am J Health Promot, 2006. **20**(6): p. 422-8.

74. Janke, K., C. Propper, and M.A. Shields, *Assaults, murders and walkers: The impact of violent crime on physical activity.* J Health Econ, 2016. **47**: p. 34-49.

75. Kaczynski, A.T. and T.D. Glover, *Talking the talk, walking the walk: examining the effect of neighbourhood walkability and social connectedness on physical activity.* J Public Health (Oxf), 2012. **34**(3): p. 382-9.

76. Kerr, Z., et al., *Changes in walking associated with perceived neighborhood safety and police-recorded crime: The multi-ethnic study of atherosclerosis.* Prev Med, 2015. **73**: p. 88-93.

77. Kneeshaw-Price, S.H., et al., *Neighborhood Crime-Related Safety and Its Relation to Children's Physical Activity.* J Urban Health, 2015. **92**(3): p. 472-89.

78. Kramer, D., et al., *Neighbourhood safety and leisure-time physical activity among Dutch adults: a multilevel perspective.* Int J Behav Nutr Phys Act, 2013. **10**: p. 11.

79. Kuo, J., et al., *Associations between family support, family intimacy, and neighborhood violence and physical activity in urban adolescent girls.* Am J Public Health, 2007. **97**(1): p. 101-3.

80. Mason, P., A. Kearns, and M. Livingston, *"Safe Going": the influence of crime rates and perceived crime and safety on walking in deprived neighbourhoods.* Soc Sci Med, 2013. **91**: p. 15-24.

81. Merom, D., et al., *Can a motivational intervention overcome an unsupportive environment for walking--findings from the Step-by-Step Study.* Ann Behav Med, 2009. **38**(2): p. 137-46.

82. Muthuri, S.K., et al., *Associations Between Parental Perceptions of the Neighborhood Environment and Childhood Physical Activity: Results from ISCOLE-Kenya.* J Phys Act Health, 2016. **13**(3): p. 333-43.

83. Nehme, E.K., et al., *Environmental Correlates of Recreational Walking in the Neighborhood.* Am J Health Promot, 2016. **30**(3): p. 139-48.

84. Peachey, A.A. and S.L. Baller, *Perceived Built Environment Characteristics of On-Campus and Off-Campus Neighborhoods Associated With Physical Activity of College Students.* J Am Coll Health, 2015: p. 0.

85. Rhodes, R.E., S.G. Brown, and C.A. McIntyre, *Integrating the perceived neighborhood environment and the theory of planned behavior when predicting walking in a Canadian adult sample.* Am J Health Promot, 2006. **21**(2): p. 110-8.

86. Robinson, A.I., F. Carnes, and N.M. Oreskovic, *Spatial analysis of crime incidence and adolescent physical activity.* Prev Med, 2016. **85**: p. 74-7.

87. Sallis, J.F., et al., *Perceived environmental predictors of physical activity over 6 months in adults: activity counseling trial.* Health Psychol, 2007. **26**(6): p. 701-9.

88. Schoeny, M.E., et al., *Barriers to physical activity as moderators of intervention effects.* Prev Med Rep, 2017. **5**: p. 57-64.

89. Siceloff, E.R., S.M. Coulon, and D.K. Wilson, *Physical activity as a mediator linking neighborhood environmental supports and obesity in African Americans in the path trial.* Health Psychol, 2014. **33**(5): p. 481-9.

90. Strath, S.J., et al., *Measured and perceived environmental characteristics are related to accelerometer defined physical activity in older adults.* Int J Behav Nutr Phys Act, 2012. **9**: p. 40.

91. Timperio, A., J. Veitch, and A. Carver, *Safety in numbers: Does perceived safety mediate associations between the neighborhood social environment and physical activity among women living in disadvantaged neighborhoods?* Prev Med, 2015.

92. Troped, P.J., et al., *Perceived built environment and physical activity in U.S. women by sprawl and region.* Am J Prev Med, 2011. **41**(5): p. 473-9.

93. Wang, Z. and C. Lee, *Site and neighborhood environments for walking among older adults.* Health Place, 2010. **16**(6): p. 1268-79.

94. Adlakha, D., et al., *"Can we walk?" Environmental supports for physical activity in India.* Prev Med, 2017. **103s**: p. S81-s89.

95. Alton, D., et al., *Relationship between walking levels and perceptions of the local neighbourhood environment.* Arch Dis Child, 2007. **92**(1): p. 29-33.

96. Andersen, L., J. Gustat, and A.B. Becker, *The Relationship Between the Social Environment and Lifestyle-Related Physical Activity in a Low-Income African American Inner-City Southern Neighborhood.* Journal of Community Health, 2015. **40**(5): p. 967-974.

97. Andrade, A.C., et al., *Social context of neighborhood and socioeconomic status on leisure-time physical activity in a Brazilian urban center: The BH Health Study.* Cad Saude Publica, 2015. **31 Suppl 1**: p. 136-47.

98. Beenackers, M.A., et al., *Why some walk and others don't: exploring interactions of perceived safety and social neighborhood factors with psychosocial cognitions.* Health Educ Res, 2013. **28**(2): p. 220-33.

99. Bringolf-Isler, B., et al., *Personal and environmental factors associated with active commuting to school in Switzerland.* Prev Med, 2008. **46**(1): p. 67-73.

100. Cerin, E., et al., *Places where preschoolers are (in)active: an observational study on Latino preschoolers and their parents using objective measures.* Int J Behav Nutr Phys Act, 2016. **13**: p. 29.

101. Chaudhury, H., et al., *Neighbourhood environment and physical activity in older adults.* Soc Sci Med, 2016. **149**: p. 104-13.

102. Chen, T.A., et al., *Features of perceived neighborhood environment associated with daily walking time or habitual exercise: differences across gender, age, and employment status in a community-dwelling population of Japan.* Environ Health Prev Med, 2013. **18**(5): p. 368-76.

103. Datar, A., N. Nicosia, and V. Shier, *Parent perceptions of neighborhood safety and children's physical activity, sedentary behavior, and obesity: evidence from a national longitudinal study.* Am J Epidemiol, 2013. **177**(10): p. 1065-73.

104. Deweese, R.S., et al., *Neighborhood perceptions and active school commuting in low-income cities.* Am J Prev Med, 2013. **45**(4): p. 393-400.

105. D'Haese, S., et al., *The association between the parental perception of the physical neighborhood environment and children's location-specific physical activity.* BMC Public Health, 2015. **15**: p. 565.

106. Ding, D., et al., *Perceived neighborhood environment and physical activity in 11 countries: do associations differ by country?* Int J Behav Nutr Phys Act, 2013. **10**: p. 57.

107. Duke, N.N., I.W. Borowsky, and S.L. Pettingell, *Parent perceptions of neighborhood: relationships with US youth physical activity and weight status.* Matern Child Health J, 2012. **16**(1): p. 149-57.

108. Echeverria, S.E., et al., *A community survey on neighborhood violence, park use, and physical activity among urban youth.* J Phys Act Health, 2014. **11**(1): p. 186-94.

109. Esteban-Cornejo, I., et al., *Parental and Adolescent Perceptions of Neighborhood Safety Related to Adolescents' Physical Activity in Their Neighborhood.* Res Q Exerc Sport, 2016. **87**(2): p. 191-9.

110. Evenson, K.R., et al., *Girls' perception of physical environmental factors and transportation: reliability and association with physical activity and active transport to school.* Int J Behav Nutr Phys Act, 2006. **3**: p. 28.

111. Gallagher, N.A., P.J. Clarke, and K.A. Gretebeck, *Gender differences in neighborhood walking in older adults.* J Aging Health, 2014. **26**(8): p. 1280-300.

112. Gay, J.L., R.P. Saunders, and M. Dowda, *The relationship of physical activity and the built environment within the context of self-determination theory.* Ann Behav Med, 2011. **42**(2): p. 188-96.

113. Handy, S.L., X. Cao, and P.L. Mokhtarian, *The causal influence of neighborhood design on physical activity within the neighborhood: evidence from Northern California.* Am J Health Promot, 2008. **22**(5): p. 350-8.

114. Heitzler, C.D., et al., *Correlates of physical activity in a national sample of children aged 9-13 years.* Prev Med, 2006. **42**(4): p. 254-60.

115. Hume, C., J. Salmon, and K. Ball, *Associations of children's perceived neighborhood environments with walking and physical activity.* Am J Health Promot, 2007. **21**(3): p. 201-7.

116. Jack, E. and G.R. McCormack, *The associations between objectively-determined and self-reported urban form characteristics and neighborhood-based walking in adults.* Int J Behav Nutr Phys Act, 2014. **11**: p. 71.

117. Kamphuis, C.B., et al., *Socioeconomic status, environmental and individual factors, and sports participation.* Med Sci Sports Exerc, 2008. **40**(1): p. 71-81.

118. Katapally, T.R. and N. Muhajarine, *Capturing the Interrelationship between Objectively Measured Physical Activity and Sedentary Behaviour in Children in the Context of Diverse Environmental Exposures.* Int J Environ Res Public Health, 2015. **12**(9): p. 10995-1011.

119. Kerr, J., et al., *Exercise aids, neighborhood safety, and physical activity in adolescents and parents.* Med Sci Sports Exerc, 2008. **40**(7): p. 1244-8.

120. Kim, H.J. and K.M. Heinrich, *Built Environment Factors Influencing Walking to School Behaviors: A Comparison between a Small and Large US City.* Front Public Health, 2016. **4**: p. 77.

121. Kramer, D., et al., *Social neighborhood environment and sports participation among Dutch adults: does sports location matter?* Scand J Med Sci Sports, 2015. **25**(2): p. 273-9.

122. Kurka, J.M., et al., *Patterns of neighborhood environment attributes in relation to children's physical activity.* Health Place, 2015. **34**: p. 164-70.

123. Lavin Fueyo, J., et al., *Neighborhood and family perceived environments associated with children's physical activity and body mass index.* Prev Med, 2016. **82**: p. 35-41.

124. Li, M., et al., *Factors associated with adolescents' physical inactivity in Xi'an City, China.* Med Sci Sports Exerc, 2006. **38**(12): p. 2075-85.

125. Li, Y., D. Kao, and T.Q. Dinh, *Correlates of neighborhood environment with walking among older Asian Americans.* J Aging Health, 2015. **27**(1): p. 17-34.

126. Liao, Y., et al., *Perceived environmental factors associated with physical activity among normal-weight and overweight Japanese men.* Int J Environ Res Public Health, 2011. **8**(4): p. 931-43.

127. Machado-Rodrigues, A.M., et al., *Parental perceptions of neighborhood environments, BMI, and active behaviors in girls aged 7-9 years.* Am J Hum Biol, 2014. **26**(5): p. 670-5.

128. Mackenbach, J.D., et al., *Exploring why residents of socioeconomically deprived neighbourhoods have less favourable perceptions of their neighbourhood environment than residents of wealthy neighbourhoods.* Obes Rev, 2016. **17 Suppl 1**: p. 42-52.

129. Maisel, J.L., *Impact of Older Adults' Neighborhood Perceptions on Walking Behavior.* J Aging Phys Act, 2016. **24**(2): p. 247-55.

130. Mota, J., et al., *Perceived neighborhood environments and physical activity in an elderly sample.* Percept Mot Skills, 2007. **104**(2): p. 438-44.

131. Mota, J., J.C. Ribeiro, and M.P. Santos, *Obese girls differences in neighbourhood perceptions, screen time and socioeconomic status according to level of physical activity.* Health Educ Res, 2009. **24**(1): p. 98-104.

132. Mota, J., et al., *Association of perceived environmental characteristics and participation in organized and non-organized physical activities of adolescents.* Pediatr Exerc Sci, 2009. **21**(2): p. 233-9.

133. Motl, R.W., et al., *Perceptions of physical and social environment variables and self-efficacy as correlates of self-reported physical activity among adolescent girls.* J Pediatr Psychol, 2007. **32**(1): p. 6-12.

134. Oluyomi, A.O., et al., *Parental safety concerns and active school commute: correlates across multiple domains in the home-to-school journey.* Int J Behav Nutr Phys Act, 2014. **11**(1): p. 32.

135. Oyeyemi, A.L., et al., *Perceived crime and traffic safety is related to physical activity among adults in Nigeria.* BMC Public Health, 2012. **12**: p. 294.

136. Oyeyemi, A.Y., O. Akinrolie, and A.L. Oyeyemi, *Health-related physical activity is associated with perception of environmental hygiene and safety among adults in low-income neighbourhoods in Nigeria.* European Journal of Physiotherapy, 2015. **17**(1): p. 45-53.

137. Piro, F.N., O. Noss, and B. Claussen, *Physical activity among elderly people in a city population: the influence of neighbourhood level violence and self perceived safety.* J Epidemiol Community Health, 2006. **60**(7): p. 626-32.

138. Rech, C.R., et al., *Personal, social and environmental correlates of physical activity in adults from Curitiba, Brazil.* Prev Med, 2014. **58**: p. 53-7.

139. Reis, R.S., et al., *Assessing participation in community-based physical activity programs in Brazil.* Med Sci Sports Exerc, 2014. **46**(1): p. 92-8.

140. Salmon, J., et al., *Are associations between the perceived home and neighbourhood environment and children's physical activity and sedentary behaviour moderated by urban/rural location?* Health Place, 2013. **24**: p. 44-53.

141. Santos, R., et al., *Physical activity and perceived environmental attributes in a sample of Portuguese adults: results from the Azorean Physical Activity and Health study.* Prev Med, 2008. **47**(1): p. 83-8.

142. Santos, M.S., et al., *Socio-demographic and perceived environmental correlates of walking in Portuguese adults--a multilevel analysis.* Health Place, 2009. **15**(4): p. 1094-9.

143. Shenassa, E.D., A. Liebhaber, and A. Ezeamama, *Perceived safety of area of residence and exercise: a pan-European study.* Am J Epidemiol, 2006. **163**(11): p. 1012-7.

144. Singh, G.K., et al., *Independent and joint effects of socioeconomic, behavioral, and neighborhood characteristics on physical inactivity and activity levels among US children and adolescents.* J Community Health, 2008. **33**(4): p. 206-16.

145. Sugiyama, T., et al., *Neighborhood environmental attributes and adults' maintenance of regular walking.* Med Sci Sports Exerc, 2015. **47**(6): p. 1204-10.

146. Tanaka, C., et al., *Conformity to the neighborhood modifies the association between recreational walking and social norms among middle-aged Japanese people.* Japan Journal of Nursing Science, 2016. **13**(4): p. 451-465.

147. Van Dyck, D., et al., *Moderating effects of age, gender and education on the associations of perceived neighborhood environment attributes with accelerometer-based physical activity: The IPEN adult study.* Health Place, 2015. **36**: p. 65-73.

148. Vanwolleghem, G., et al., *Which Socio-Ecological Factors Associate with a Switch to or Maintenance of Active and Passive Transport during the Transition from Primary to Secondary School?* PLoS One, 2016. **11**(5): p. e0156531.

149. Verhoeven, H., et al., *Psychosocial and Environmental Correlates of Walking, Cycling, Public Transport and Passive Transport to Various Destinations in Flemish Older Adolescents.* PLoS One, 2016. **11**(1): p. e0147128.

150. Weber Corseuil, M., et al., *Safety from crime and physical activity among older adults: a population-based study in Brazil.* J Environ Public Health, 2012. **2012**: p. 641010.

151. Weir, L.A., D. Etelson, and D.A. Brand, *Parents' perceptions of neighborhood safety and children's physical activity.* Prev Med, 2006. **43**(3): p. 212-7.

152. Zhu, X., B. Arch, and C. Lee, *Personal, social, and environmental correlates of walking to school behaviors: case study in Austin, Texas.* ScientificWorldJournal, 2008. **8**: p. 859-72.

153. Datar, A., et al., *Neighborhood Environment and Children's Physical Activity and Body Mass Index: Evidence from Military Personnel Installation Assignments.* Child Obes, 2015.

154. Eichinger, M., et al., *How are physical activity behaviors and cardiovascular risk factors associated with characteristics of the built and social residential environment?* PLoS One, 2015. **10**(6): p. e0126010.

155. Inoue, S., et al., *Perceived neighborhood environment and walking for specific purposes among elderly Japanese.* J Epidemiol, 2011. **21**(6): p. 481-90.

156. Pelclova, J., K. Fromel, and R. Cuberek, *Gender-specific associations between perceived neighbourhood walkability and meeting walking recommendations when walking for transport and recreation for Czech inhabitants over 50 years of age.* Int J Environ Res Public Health, 2014. **11**(1): p. 527-36.

157. Perez, L.G., et al., *Interactions between individual and perceived environmental factors on Latinas' physical activity.* J Public Health (Oxf), 2017. **39**(2): p. e10-e18.

158. Tappe, K.A., et al., *Children's physical activity and parents' perception of the neighborhood environment: neighborhood impact on kids study.* Int J Behav Nutr Phys Act, 2013. **10**: p. 39.

159. Carver, A., et al., *Are children and adolescents less active if parents restrict their physical activity and active transport due to perceived risk?* Soc Sci Med, 2010. **70**(11): p. 1799-805.

160. Garrett, N., P.J. Schluter, and G. Schofield, *Physical activity profiles and perceived environmental determinants in New Zealand: a national cross-sectional study.* J Phys Act Health, 2012. **9**(3): p. 367-77.

161. Jilcott Pitts, S.B., et al., *Associations between neighborhood-level factors related to a healthful lifestyle and dietary intake, physical activity, and support for obesity prevention polices among rural adults.* J Community Health, 2015. **40**(2): p. 276-84.

162. Loch, M.R., et al., *Relationship between social capital indicators and lifestyle in Brazilian adults.* Cadernos de Saúde Pública, 2015. **31**: p. 1636-1647.

163. Mackenbach, J.D., et al., *Neighbourhood social capital: measurement issues and associations with health outcomes.* Obes Rev, 2016. **17 Suppl 1**: p. 96-107.

164. Marlier, M., et al., *Interrelation of Sport Participation, Physical Activity, Social Capital and Mental Health in Disadvantaged Communities: A SEM-Analysis.* PLoS One, 2015. **10**(10): p. e0140196.

165. Remmers, T., et al., *Moderators of the longitudinal relationship between the perceived physical environment and outside play in children: the KOALA birth cohort study.* Int J Behav Nutr Phys Act, 2014. **11**(1): p. 150.

166. Ueshima, K., et al., *Does social capital promote physical activity? A population-based study in Japan.* PLoS One, 2010. **5**(8): p. e12135.

167. Davison, K.K., et al., *Associations among social capital, parenting for active lifestyles, and youth physical activity in rural families living in upstate New York.* Soc Sci Med, 2012. **75**(8): p. 1488-96.

168. Strong, L.L., et al., *Associations of perceived neighborhood physical and social environments with physical activity and television viewing in African-American men and women.* Am J Health Promot, 2013. **27**(6): p. 401-9.

169. King, D., *Neighborhood and individual factors in activity in older adults: results from the neighborhood and senior health study.* J Aging Phys Act, 2008. **16**(2): p. 144-70.

170. Samuel, L.J., et al., *Social engagement and chronic disease risk behaviors: The Multi-Ethnic Study of Atherosclerosis.* Prev Med, 2015. **71**: p. 61-6.

171. Gao, J., *Association between social and built environments and leisure-time physical activity among Chinese older adults--a multilevel analysis.* BMC Public Health, 2015. **15**: p. 1317-28.

172. Mendes de Leon, C.F., et al., *Neighborhood social cohesion and disorder in relation to walking in community-dwelling older adults: a multilevel analysis.* J Aging Health, 2009. **21**(1): p. 155-71.

173. Salahuddin, M., et al., *Does Parents' Social Cohesion Influence Their Perception of Neighborhood Safety and Their Children's Active Commuting to and From School?* J Phys Act Health, 2016. **13**(12): p. 1301-1309.

174. Carver, A., A.F. Timperio, and D.A. Crawford, *Bicycles gathering dust rather than raising dust--Prevalence and predictors of cycling among Australian schoolchildren.* J Sci Med Sport, 2015. **18**(5): p. 540-4.

175. Van Cauwenberg, J., et al., *Relationships between the perceived neighborhood social environment and walking for transportation among older adults.* Soc Sci Med, 2014. **104**: p. 23-30.

176. Macniven, R., et al., *Physical activity, healthy lifestyle behaviors, neighborhood environment characteristics and social support among Australian Aboriginal and non-Aboriginal adults.* Prev Med Rep, 2016. **3**: p. 203-10.

177. McDonald, N.C., *The effect of objectively measured crime on walking in minority adults.* Am J Health Promot, 2008. **22**(6): p. 433-6.

178. Wen, M. and X. Zhang, *Contextual effects of built and social environments of urban neighborhoods on exercise: a multilevel study in Chicago.* Am J Health Promot, 2009. **23**(4): p. 247-54.

179. Amorim, T.C., M.R. Azevedo, and P.C. Hallal, *Physical activity levels according to physical and social environmental factors in a sample of adults living in South Brazil.* J Phys Act Health, 2010. **7 Suppl 2**: p. S204-12.

180. Halbert, C.H., et al., *Collective efficacy and obesity-related health behaviors in a community sample of African Americans.* J Community Health, 2014. **39**(1): p. 124-31.

181. Cleland, V.J., A. Timperio, and D. Crawford, *Are perceptions of the physical and social environment associated with mothers' walking for leisure and for transport? A longitudinal study.* Prev Med, 2008. **47**(2): p. 188-93.
